# Non-enzymatic oligonucleotide ligation in coacervate protocells sustains compartment-content coupling

**DOI:** 10.1038/s41467-023-38163-8

**Published:** 2023-05-09

**Authors:** Tommaso P. Fraccia, Nicolas Martin

**Affiliations:** 1 Institut Pierre-Gilles de Gennes, Chimie Biologie et Innovation, UMR 8231, ESPCI Paris, PSL University, CNRS, 6 rue Jean Calvin, 75005 Paris, France; 2 Department of Pharmacological and Biomolecular Sciences, University of Milano, 20133 Milano, Italy; 3 Univ. Bordeaux, CNRS, Centre de Recherche Paul Pascal, UMR 5031, 115 avenue du Dr. Schweitzer, 33600 Pessac, France

**Keywords:** Self-assembly, Origin of life, Liquid crystals, Bioinspired materials

## Abstract

Modern cells are complex chemical compartments tightly regulated by an underlying DNA-encoded program. Achieving a form of coupling between molecular content, chemical reactions, and chassis in synthetic compartments represents a key step to the assembly of evolvable protocells but remains challenging. Here, we design coacervate droplets that promote non-enzymatic oligonucleotide polymerization and that restructure as a result of the reaction dynamics. More specifically, we rationally exploit complexation between end-reactive oligonucleotides able to stack into long physical polymers and a cationic azobenzene photoswitch to produce three different phases—soft solids, liquid crystalline or isotropic coacervates droplets—each of them having a different impact on the reaction efficiency. Dynamical modulation of coacervate assembly and dissolution via *trans*-*cis* azobenzene photo-isomerization is used to demonstrate cycles of light-actuated oligonucleotide ligation. Remarkably, changes in the population of polynucleotides during polymerization induce phase transitions due to length-based DNA self-sorting to produce multiphase coacervates. Overall, by combining a tight reaction-structure coupling and environmental responsiveness, our reactive coacervates provide a general route to the non-enzymatic synthesis of polynucleotides and pave the way to the emergence of a primitive compartment-content coupling in membrane-free protocells.

## Abstract (web summary)

Achieving a form of coupling between molecular content, chemical reactions, and chassis in synthetic compartments represents a key step to the assembly of evolvable protocells but remains challenging. Here, the authors design coacervate droplets that promote non-enzymatic oligonucleotide polymerization and that restructure as a result of the reaction dynamics.

## Introduction

Living cells are sophisticated compartmentalized chemical systems. From that perspective, the bottom-up assembly of chemically reactive soft micro-compartments represents a first step to the de novo construction of primitive life-like systems^1,2^. Due to their membranous structure, lipid-bounded vesicles have been extensively studied as rudimentary cell-like compartments able to host various reactions^1^, from prebiotically relevant templated nucleotide condensation^3^ to gene-directed cell-free protein expression^4^. Membrane-free droplets produced by complex coacervation between oppositely charged polyions have recently received increasing attention as viable alternative protocells^5–7^ and surrogates of intracellular biomolecular condensates^8^. Coacervates assemble from a diversity of species, including prebiotically plausible molecules^6,9^, guide the interfacial assembly of lipid membranes^10,11^, can organize into multiple sub-compartments^12–14^ and be formed and dissolved in response to physical and chemical stimuli^15–21^. Owing to their lack of a membrane, liquid-like nature, low internal polarity, and high local charge density^8^, coacervate microdroplets also spontaneously uptake and accumulate various guest solutes by partitioning^22,23^, including nucleotides, RNA, and divalent ions^24^. This local up-concentration has been shown to accelerate or enhance otherwise slow or inefficient enzyme reactions^25–27^ and has been exploited to design coacervates capable of supporting ribozyme catalysis^28,29^, offering a new hypothesis for the RNA world scenario. Yet, whether coacervate droplets can favor or guide simpler chemical reactions without the need for complex catalytic macromolecules and machinery remains elusive, as non-enzymatic reactions in coacervate droplets are still largely unexplored^30,31^. In a recent study, the lower internal polarity of peptide coacervates, in addition to increased local concentration effects, have been suggested to play a role in promoting aldol reactions and hydrazone formation^30^. However, non-enzymatic polymerization reactions, which are critically relevant to the abiotic emergence of functional biopolymers such as polynucleotides^3,32–35^, have not been demonstrated to date in complex coacervate droplets.

Carbodiimide-activated oligonucleotide ligation has recently been achieved in concentrated liquid crystalline DNA phases produced by segregative liquid-liquid phase separation (LLPS) with the uncharged poly(ethylene glycol) (PEG) polymer^36–39^. Unlike coacervates, these aqueous two-phase systems only form at elevated concentrations of polymer species (typically 100–400 g L^−1^ vs. 1–2 g L^−1^ for coacervates), which limits their relevance as protocells and significantly differ in composition and properties from complex coacervates, in which high concentrations of polyions can drastically affect the hosted reactions. For instance, aqueous two-phase systems formed with the neutral PEG and dextran polymers were shown to enhance ribozyme catalysis due to increased local concentrations^40^, yet, the same reaction was drastically reduced in complex coacervates due to undesirable ion pairing with polycations, which required careful optimization of the coacervate composition^28,29^.

To elucidate the interplay between associative LLPS and oligonucleotide polymerization, coacervates with different and controllable internal organizations are highly desirable. Liquid crystalline coacervates droplets assembled from oligonucleotides and poly-Lysine have recently been reported^41–43^ but are not compatible with condensation reactions due to the cross-reactivity of polyamines with carbodiimide. We also recently designed light-responsive DNA coacervates assembled from random polynucleotide sequences and a cationic photoswitch on which the positive charge was provided by a non-reactive quaternary ammonium^15,44^. Building upon these observations, we here aimed at assembling reactive oligonucleotide coacervates with controllable internal order to rationalize the impact of the latter on non-enzymatic DNA ligation. Since the formation and stability of complex coacervates are intrinsically related to the length, flexibility, or charge density of their scaffold polyions^45,46^, we also intended to investigate the effect of in situ polynucleotide elongation on the properties of the coacervate droplets. Dynamically coupling in situ chemical reactivity and coacervate properties would indeed provide invaluable insights into the interplay between abiotic reactions and phase separation^47^.

Here, we show that mixtures of end-stacking oligonucleotides and a molecular azobenzene cation orthogonal to carbodiimide chemistry produce three different phases depending on the ionic strength or light–soft solids, liquid crystalline or isotropic coacervates droplets—that significantly enhance chemical oligonucleotide ligation, with a marked phase-dependent reaction efficiency. We take advantage of light-mediated *trans*-*cis* azobenzene photo-isomerization to dynamically modulate the formation and dissolution of coacervate droplets and achieve light-actuated DNA ligation as a demonstration of temporal control over polynucleotide elongation in periodically changing environmental conditions. Excitingly, we last observe that changes in the length distribution of polynucleotides during in situ polymerization induce phase transitions of or within the coacervate droplets that dramatically alter their physical properties and structure. Overall, although the design of our system does not solely involve prebiotically relevant components, our complex coacervation platform offers a general approach to the non-enzymatic synthesis of polynucleotides from shorter oligomers by combining a tight reaction-structure relationship and environmental responsiveness and paves the way to the emergence of compartment-content coupling in membrane-free protocells.

## Results

### Phase behavior of end-to-end stacking oligonucleotides and azobenzene cations with salt and light

**Fig. 1:**
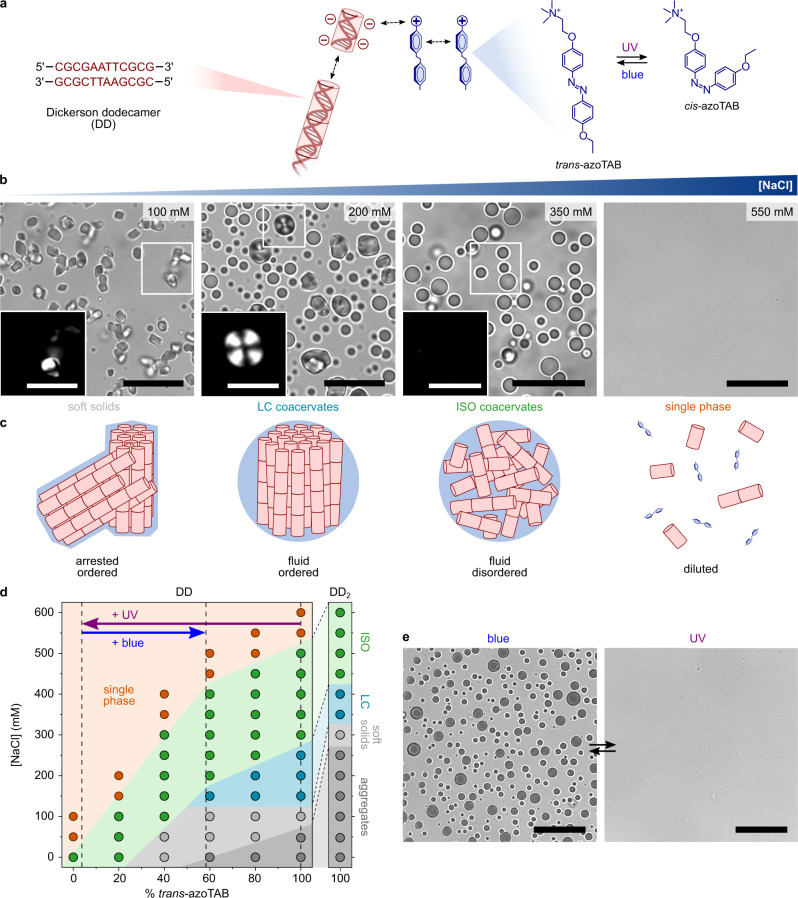
Salt- and light-dependent phase behavior of end-interacting oligonucleotides and azobenzene cations. **a** Sequence and hybridization of the self-complementary DNA Dickerson dodecamer (DD) used in the study (left), chemical structure of *trans*-azoTAB and reversible photo-isomerization to *cis*-azoTAB (right), and schematic representation of interactions involved in DD/*trans*-azoTAB complexation (middle). **b** Bright-field optical microscopy images of charge-balanced mixtures of DD (5 mM nucleobase concentration) and *trans*-azoTAB (5 mM) prepared at different NaCl concentrations, as indicated, showing the formation of irregular soft solids (100 mM NaCl), liquid crystal (LC) coacervate droplets (200 mM NaCl), isotropic (ISO) coacervate droplets (350 mM NaCl), or absence of phase separation (550 mM NaCl). Scale bars, 20 μm. Insets show zoomed images of the white squared areas under 90° crossed polarizers. Scale bars, 10 μm. **c** Schematic views of the self-assembled complexes produced at the different salt concentrations shown in (**b**). **d** Equilibrium phase diagram of charge-balanced mixtures of DD or DD_2_ (5 mM nucleobase concentration) and azoTAB (5 mM total concentration) at varying *trans*:*cis* fraction (for DD) and at 100% *trans*-azoTAB (for DD_2_) and at varying NaCl concentration. The complexes produced for each condition were characterized by optical microscopy (as shown in Supplementary Fig. 7) and classified as solid-like aggregates (dark gray), soft solids (light gray), LC coacervates (cyan), ISO coacervates (green), and no phase separation (orange). The fractions of *cis*- and *trans*-azoTAB produced under UV or blue light are also indicated (dashed vertical lines). **e** Optical microscopy images of DD/*trans*-azoTAB ISO coacervate droplets prepared at 350 mM NaCl under blue light or UV light, as indicated. Scale bars, 20 μm.

Our experimental coacervate system was designed to exhibit a diversity of controllable phases while being orthogonal to carbodiimide chemistry. We chose to work with the light-responsive azobenzene cation *trans*-azobenzene trimethylammonium bromide (*trans*-azoTAB, Fig. 1a) that we recently used to produce photoswitchable DNA coacervates with polydisperse polynucleotides^15^. The cationic charge on *trans*-azoTAB is provided by a non-reactive quaternary ammonium group to prevent any interference with the ligation reaction.

We first investigated the salt-dependent phase behavior of charge-balanced mixtures of *trans*-azoTAB (5 mM) and the self-complementary DNA Dickerson dodecamer sequence (DD, 5’-CGCGAATTCGCG-3’, 1.5 mg mL^−^^1^, 5 mM nucleobase concentration), which hybridizes into blunt-end duplexes capable of liquid crystal ordering at high concentration^36,37,48^ (>300 mg mL^−^^1^, Fig. 1a). Optical microscopy images revealed the formation of solid-like precipitates in pure water (Supplementary Fig. 1), while soft solids or liquid-like microdroplets were observed when the ionic strength was gradually increased (Fig. 1b and Supplementary Fig. 2) due to the weakening of attractive electrostatic interactions, as reported for other coacervates^41,43,49^. Interestingly, droplets prepared at intermediate salt concentrations exhibited a strong birefringence under crossed polarizers, with textures typical of columnar liquid crystalline phase (Fig. 1b, 200 mM, and Supplementary Fig. 3), while they appeared isotropic (ISO) at higher ionic strength (Fig. 1b, 350 mM). These observations were in line with the salt-dependent formation of ISO or LC coacervate droplets recently reported in DD/poly-L-Lysine mixtures^41,42^, where LC ordering was attributed to the supramolecular assembly of DD duplexes into long linear aggregates via end-to-end stacking. Here, inhibition of end-to-end interactions by the addition of TT overhangs at the 3’-terminus of the DD sequence prevented the formation of LC droplets (Supplementary Fig. 4), which confirmed that LC ordering required the supramolecular assembly of DD duplexes into long linear aggregates via end-to-end stacking (Fig. 1c). This observation also revealed that coacervate phases were intrinsically dependent on the oligonucleotide sequence. Fluorescence recovery after photobleaching confirmed the increase in the internal fluidity of the phases at increasing ionic strength (Supplementary Fig. 5). Coacervate microdroplets disassembled above a critical salt concentration (Fig. 1b and Supplementary Fig. 1, [NaCl] > 520 mM), indicating that electrostatic interactions were a driving force for DD/*trans*-azoTAB complexation. The addition of the 1,6-hexanediol aliphatic alcohol or the water-miscible organic solvent DMSO also resulted in coacervate disassembly (Supplementary Fig. 6), pointing to the additional role of weak hydrophobic interactions for coacervate formation, such as azobenzene π-π self-stacking and *trans*-azoTAB intercalation between DNA base pairs, as reported previously^15^.

We also explored the influence of the azoTAB *trans*:*cis* molar ratio on the phases produced with DD at different salt concentrations. Equilibrium phase behavior studies revealed that using lower fractions of *trans*-azoTAB favored the formation of ISO coacervates at lower ionic strength in place of soft solids or LC coacervates, and resulted in lower critical salt concentrations for coacervate dissolution (Fig. 1d and Supplementary Fig. 7). These observations suggested that *cis*-azoTAB had a weaker interaction strength for DD compared to *trans*-azoTAB, as previously observed with longer double-stranded DNA (dsDNA)^15,50–52^, which opened the possibility to navigate through the DD/azoTAB phases at fixed salt concentration using light (Fig. 1d). Indeed, when exposed to UV light, *trans*-azoTAB quantitatively isomerizes into *cis*-azoTAB, a process that can be reversed with visible light (Supplementary Fig. 8). As a result, we observed that LC and ISO coacervate microdroplets readily dissolved in a few seconds when exposed to UV light due to quantitative *trans*-*cis* azoTAB photo-isomerization, and reassembled into ISO droplets under blue light due to partial *cis*-*trans* azoTAB reverse photo-isomerization (Fig. 1e, Supplementary Fig. 9 and Supplementary Movies 1, 2). This process was reversible and could be repeated several times without any apparent fatigue (Supplementary Fig. 10). In comparison, salt-free solid-like precipitates transformed into liquid-like ISO coacervate droplets under UV light but did not dissolve (Supplementary Fig. 9), as expected from the equilibrium phase diagram and similar to recently reported photo-controllable phase transitions in arylazopyrazole-conjugated oligonucleotide coacervates^53^.

Taken together, our observations show that charge-balanced mixtures of a self-complementary DNA dodecamer and *trans*-azoTAB produce ordered LC coacervate microdroplets at intermediate salt concentrations, which is attributed to the physical linear aggregation of blunt-ends oligonucleotides into longer stiff polynucleotides, together with possible contributions from azobenzene π-π self-stacking^15^ (Fig. 1a, c). In comparison, an isotropic coacervate phase is produced at higher ionic strength, presumably associated with disordered monomeric or short repeats of DD duplexes bridged together by *trans*-azoTAB (Fig. 1c). This behavior is in line with the known concentration dependence for the reversible linear aggregation of end-to-end interacting oligomeric DNA duplexes, which undergoes a sharp discontinuous increase at the ISO-LC transition^54^. By changing the fraction of *trans*- and *cis*-azoTAB, light can also be used to modulate the internal organization of DD phases and ultimately induce coacervate dissolution.

### Non-enzymatic oligonucleotide ligation depends on the internal coacervate structure

Quantitative measurements of the oligonucleotide concentration in the LC and ISO coacervate droplets revealed that ~90 mol% of DD strands were sequestered within the coacervate phase, corresponding to a local oligonucleotide concentration of ~480 ± 190 mg mL^−^^1^ in the ISO microdroplets (Supplementary Note 1). In comparison, the oligonucleotide concentration in the supernatant was 0.72 ± 0.3 mg mL^−^^1^, so a ~670-fold enhancement of DD local concentration in the ISO coacervates compared to the supernatant was observed. We sought to utilize this strong enrichment of DNA oligomers within coacervate phases to achieve in situ non-enzymatic oligonucleotide polymerization and study the influence of each phase on the ligation reaction. For this purpose, DD strands were functionalized with a 3’ terminal phosphate group to produce end-reactive DDp oligonucleotides (5’-CGCGAATTCGCG-*p*-3’) able to undergo covalent ligation, via a phosphodiester bond, upon activation with the water-soluble coupling agent 1-ethyl-3-(3-dimethylaminopropyl) carbodiimide hydrochloride^38^ (EDC, Supplementary Fig. 11). Due to its sensitivity to hydrolysis, a large molar excess of EDC compared to the reactive phosphate groups is typically required^38,39^. Here, the contribution of EDC salt to the total ionic strength of the solution produced a similar solid-to-liquid transition as NaCl and was therefore taken into account to produce the desired oligonucleotide/*trans*-azoTAB assemblies (Supplementary Fig. 12).

**Fig. 2:**
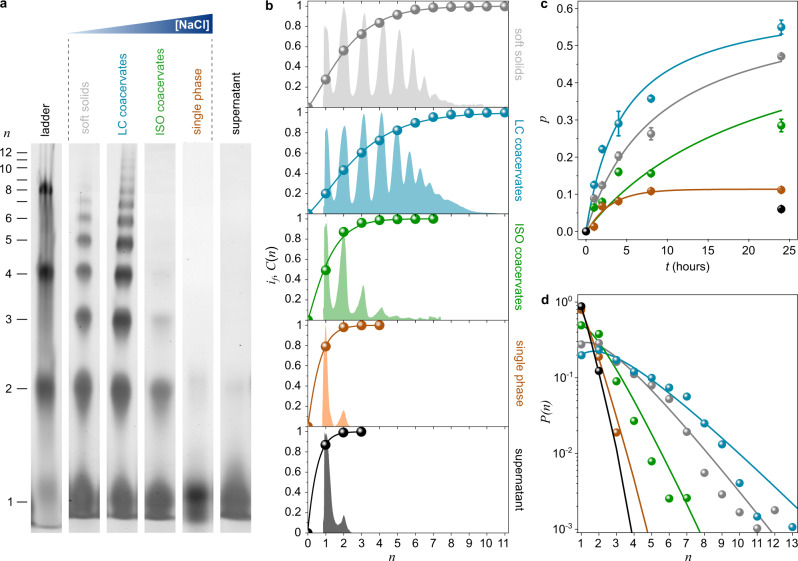
Oligonucleotide ligation depends on the nature of the DDp/*trans*-azoTAB phase. **a** Cropped gel images of a 15% PAGE run of the ligation product obtained after 24 h DDp/*trans*-azoTAB solutions (equimolar charge mixtures, 10 mM total charge concentration) in different phases, as labeled (soft solids: 80 mM EDC; LC droplets: 100 mM NaCl + 80 mM EDC; ISO droplets: 200 mM NaCl + 80 mM EDC; single phase: 400 mM NaCl + 80 mM EDC; supernatant obtained from centrifuged LC droplets). Ladder contains mixed 12-, 24-, 48- and 96-mer DNA oligomers derived from the repetition of the 12-mer DD sequence. **b** Fluorescence intensity (*i*_*f*_, colored areas), extracted from gel images shown in (**a**) and cumulative weight distribution (*C*(*n*), colored dots) plotted as a function of the degree of polymerization, *n*, derived from rescaling (see Supplementary Note 3). Solid lines show fits of *C*(*n*) data points using Flory’s theory for linear polymer condensation (see Supplementary Note 2). **c** Time-dependent evolution of polymerization yield, *p*, for the different solutions prepared as in (**a**) (gray: soft solids, cyan: LC coacervates, green: ISO coacervates, orange: single phase, black: supernatant). Yields were extracted by analyzing samples from the same experiment (*n* = 1) run in the same PAGE gel (except for single phase, whose gel was run in parallel) shown in Supplementary Fig. 13 using two fitting procedures, as detailed in Supplementary Note 3, and are reported as mean ± SD of the two values obtained from fits. Error bars thus represent errors associated with uncertainties in the fits used to extract the reaction yields. Solid lines are fits using a catalyzed step-growth polymerization model to estimate the characteristic reaction time, *τ* (see Supplementary Note 4). **d** Weight fraction distributions, *P*(*n*), after 24 h of ligation reaction in the different solutions prepared as in (**a**) (black: supernatant, gray: soft solids, cyan: LC coacervates, green: ISO coacervates, orange: single phase). Solid lines show fits of *P*(*n*) data points using Flory’s theory for linear polymer condensation (see Supplementary Note 2). Source data are provided as Source Data files.

The outcome of the ligation reaction was first monitored after 24 h in four representative DDp/*trans*-azoTAB samples prepared at varying NaCl but fixed EDC concentrations (soft solids: 80 mM EDC; LC coacervates: 100 mM NaCl + 80 mM EDC; ISO coacervates: 200 mM NaCl + 80 mM EDC; single phase: 400 mM NaCl + 80 mM EDC) using polyacrylamide gel electrophoresis (PAGE), and compared to the supernatant solution of the LC sample. The dense coacervate phase and supernatant were separated by centrifugation before PAGE analysis (see “Methods”). Our results revealed that oligonucleotide ligation occurred more efficiently in DDp-rich phases (soft solids and both types of liquid-like coacervates), as manifested by the appearance of a large distribution of oligomer bands in the polyacrylamide gels, compared to the diluted phases (single phase and supernatant) where it was very limited (Fig. 2a and Supplementary Fig. 13). Notably, the maximum degree of polymerization, *n*_*max*_, observed in the gels was significantly higher in DDp-rich phases (*n*_*max, soft solids*_ = 9; *n*_*max, LC droplets*_ = 12; *n*_*max, ISO droplets*_ = 6) compared to the single phase (*n*_*max, single phase*_ = 2) and dilute supernatant solution (*n*_*max, supernatant*_ = 2). More extensive investigations confirmed that ligation was also ineffective in samples prepared at higher salt (NaCl + EDC) concentrations (Supplementary Fig. 14), as expected from the lower coacervate volume phase produced near the critical coacervation salt concentration. We attributed such differences in reaction efficiency mainly to differences in the nature of DDp/*trans*-azoTAB phases since NaCl addition has been shown to have minimal effect on EDC-activated DNA ligation^55^.

Fluorescence intensity profiles of the gel scans were analyzed more quantitatively by exploiting the approximate proportionality between electrophoretic mobility and the logarithm of chain length^38,56^ (Fig. 2b). The cumulative weight fraction distribution, *C*(*n*), extracted from these profiles was well described by a simple Flory model for linear polymer condensation^57^ (Fig. 2b and Supplementary Note 2), despite uncertainties in the quantitative information carried by the fluorescence profiles of the gels. This model allowed us to quantify and compare the polymerization yield, *p*, and the mean degree of polymerization, *<n*>, for the different conditions tested (Supplementary Note 3). We observed that both the polymerization yield and average degree of polymerization were the highest for LC coacervate droplets (*p* = 0.55 ± 0.01, *<n*> = 3.40 ± 0.10), then decreased for soft solids (*p* = 0.47 ± 0.01, *<n*> = 2.77 ± 0.07) and ISO coacervate droplets (*p* = 0.29 ± 0.01, *<n*> = 1.82 ± 0.04), and were the lowest for the dilute phase (*p* = 0.11 ± 0.01, *<n*> = 1.25 ± 0.01) and the supernatant solution (*p* = 0.06 ± 0.01, *<n*> = 1.13 ± 0.02). The same tendency for *p* and *<n*> was observed at different reaction times (Fig. 2c and Supplementary Fig. 15). The characteristic reaction time, *τ*, extracted from fitting of *p* vs. time using a catalyzed step-growth polymerization model (Supplementary Note 4) was 9.0 ± 0.7 h, 4.5 ± 0.9 h, and 22 ± 4 h for soft solids, LC coacervates, and ISO droplets, respectively, corresponding to a 2- or 5-fold acceleration in LC coacervates compared to soft solids or ISO droplets, respectively. In addition, the products distribution gradually moved toward longer DNA sequences as a function of time while the fraction of unreacted DDp oligonucleotides (*n* = 1) decreased (Supplementary Fig. 16) so that different DNA populations were obtained after 24 h in the different DDp-rich phases (Fig. 2d). On the contrary, in the dilute single phase the reaction produced only dimeric products, whose formation took place at early stages (*τ* = 2.9 ± 0.9 h) due to non-zero probability of a 5’-3’ covalent bonding of an activated oligonucleotide with its hybridized strand.

Overall, these analyses confirmed that DD ligation was most enhanced in LC coacervates, followed by soft solids and ISO droplets. The enhanced reaction efficiency in the dense complexes compared to the supernatant was attributed to the higher local concentration of oligonucleotides in the condensates. Additional mechanisms contributing to the increased ligation efficiency in LC coacervates compared to soft solids or ISO droplets included stabilization of end-to-end supramolecular association of DNA oligonucleotides into organized repeats characteristic of the already ligated bases^54,58^, maintenance of stable proximity of the reacting termini^59^, and provision by the LC phase of a still fluid environment for transport and reaction^38^. Importantly, ligation experiments in coacervates assembled with non-stacking TT-terminated reactive DD duplexes (TT-DDp, Supplementary Table 1), which are unable to form LC droplets due to the non-pairing dangling ends (Supplementary Fig. 4), showed poor reaction efficiency (Supplementary Fig. 17). In comparison, sticky end CG-terminated duplexes (12-CG, Supplementary Table 1), which end-to-end stacking propensity is favored by base pairing, showed a similar coacervation and ligation behavior as DD strands (Supplementary Fig. 18). These results indicated that ligation enhancement seemed to depend on the capacity of DNA duplexes to undergo linear aggregation, which we attributed to the associated proximity of the reactive groups. It is also worth noting that reaction yields in LC droplets were constantly higher than those observed in soft solids (Fig. 2c), confirming that the suppressed fluidity in the latter affected EDC diffusion and DDp reorganization, in turn reducing ligation efficiency, despite the likely higher concentration of oligonucleotides in such low-salt phase (Supplementary Note 1). Our results also reveal that DD oligonucleotide ligation was almost as effective in LC coacervate droplets as in segregated pure LC DNA phases^38^, suggesting that the reaction was not disrupted by ion-pairing with azobenzene cations.

### Oligonucleotide ligation is reversibly controlled with light

**Fig. 3:**
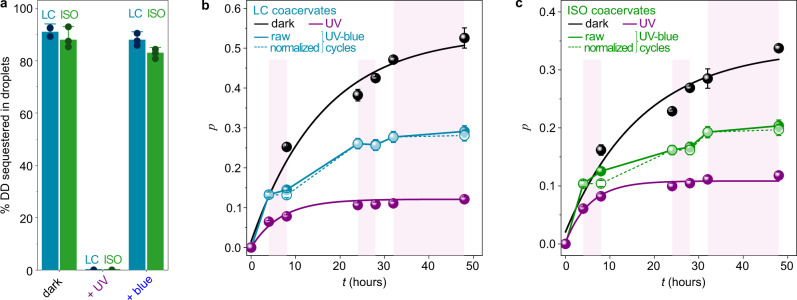
Oligonucleotide ligation is reversibly controlled with light. **a** Molar fraction of DD strands sequestered in ISO and LC coacervate droplets in the dark, under UV light, and under blue light (after exposure to UV light). The average value of three independent experiments (shown as black dots) is reported. Error bars represent the associated standard deviation. **b**, **c** Polymerization yield, *p*, calculated from fitted product distributions (see Supplementary Notes 2 and 3) for reactive LC (**b**, 100 mM NaCl + 80 mM EDC) and ISO (**c**, 200 mM NaCl + 80 mM EDC) coacervate droplets kept in the dark (black dots) or under constant UV light (purple dots), or exposed to cycles of UV and blue light (cyan or green dots, solid line; purple regions show when UV light was on). Normalized data for UV/blue cycles (open dots, dotted line) was obtained by subtracting the contribution of ligation occurring during UV exposure measured in dilute samples constantly exposed to UV light. Yields were extracted by analyzing samples from the same experiment (*n* = 1) run in the same PAGE gel (except for constant UV exposure, whose gel was run in parallel) shown in Supplementary Fig. 19 using two fitting procedures, as detailed in Supplementary Note 3, and are reported as mean ± SD of the two values obtained from fits. Error bars thus represent errors associated with uncertainties in the fits used to extract the reaction yields. Solid lines for dark and UV conditions are fits using a catalyzed step-growth polymerization model; other solid lines are guides to the eye. Source data are provided as Source Data file.

UV light-mediated dissolution of LC and ISO coacervate droplets resulted in the quantitative release of DD strands in the aqueous solution to give an oligonucleotide concentration of 1.50 ± 0.04 mg mL^−^^1^ (Fig. 3a) so that a ~320-fold enhancement of DD local concentration in the dark-adapted ISO coacervates compared to the UV-adapted homogeneous solution was observed. Reversibly, exposure to blue light led to the recapture of more than 80 mol% of oligonucleotides within the reassembled coacervate phase (Fig. 3a). These photoswitchable coacervates thus appeared as a viable platform to reversibly up- or downregulate the local concentration of oligonucleotides using light, and in turn modulate the ligation reaction.

We therefore proceeded to demonstrate light-actuated ON/OFF oligonucleotide ligation. LC and ISO DDp/*trans*-azoTAB coacervate droplets prepared under reactive conditions were either stored in the dark for 48 h, continuously irradiated with UV light, or exposed to cycles of UV and blue light, and polyacrylamide gel scans were performed over time to extract the reaction yield, *p*, and average, <*n*> degree of polymerization (Supplementary Fig. 19). Results revealed that UV light-exposed dilute DDp/*cis*-azoTAB samples exhibited a much lower oligonucleotide ligation efficiency compared to dark-adapted samples (Fig. 3b, c), and similar to that of reaction performed in diluted single phase at higher salt (Fig. 2c). More specifically, we observed that *p*, and <*n*> increased monotonously and rapidly for samples kept in the dark, with a stronger increase for LC coacervates compared to ISO droplets, as expected from the enhanced ligation efficiency associated to LC ordering, while UV-exposed samples showed a poor ligation efficiency (Fig. 3b, c and Supplementary Fig. 20). Remarkably, for samples exposed to cycles of UV and blue light, the reaction yield and degree of polymerization only increased under blue light conditions but remained almost constant during UV light exposure (Fig. 3b, c and Supplementary Fig. 20). These results therefore demonstrate that DD oligonucleotide ligation could be periodically modulated with light based on the reversible dissolution of coacervate droplets and the resulting change in the local oligonucleotide concentration. Interestingly, such an environmentally responsive transient compartmentalization could be particularly useful in overcoming the parasite problem^60^.

### Oligonucleotide elongation produces phase transitions and multiphase coacervate droplets

**Fig. 4:**
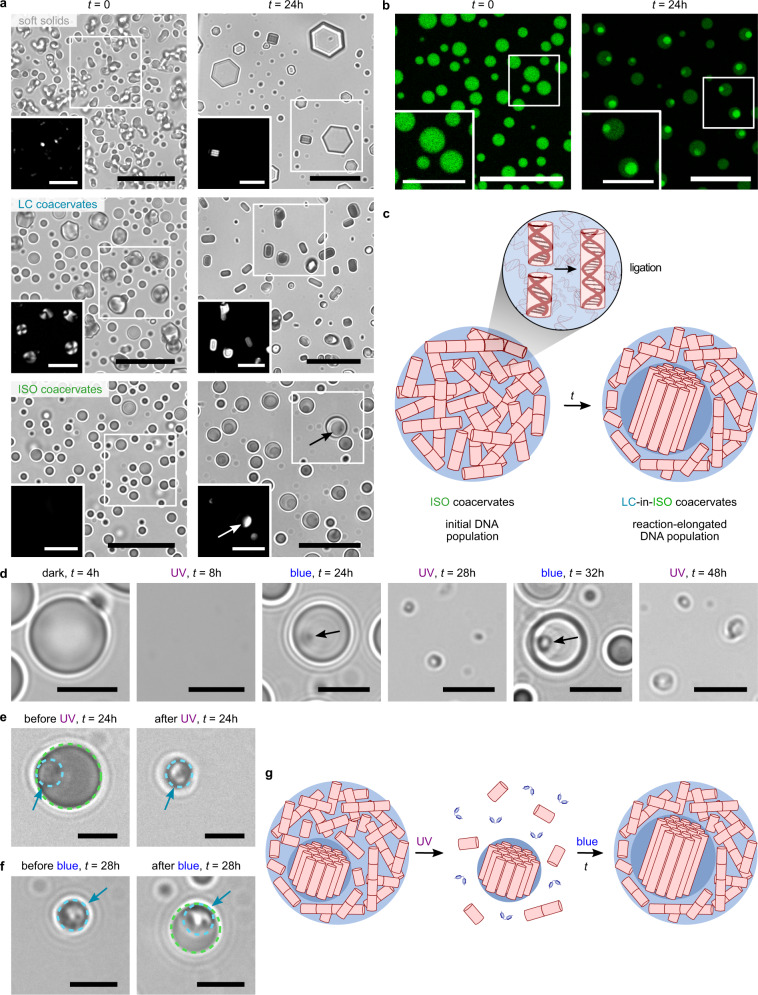
Structural transformation of coacervates during DD oligonucleotide ligation. **a** Bright-field optical microscopy images of charged-balanced reactive mixtures of DDp (5 mM nucleobase concentration) and *trans*-azoTAB (5 mM) prepared at different total ionic strength (soft solids: 80 mM EDC (top); LC droplets: 100 mM NaCl + 80 mM EDC (middle); ISO droplets: 200 mM NaCl + 80 mM EDC (bottom)) at *t* = 0 and after 24 h, as labeled. Scale bars, 20 μm. Insets show the white squared areas under 90° crossed polarizers. Scale bars, 10 μm. **b** Confocal fluorescence microscopy images of reactive ISO coacervate droplets (5 mM DDp nucleobase concentration, 5 mM *trans*-azoTAB, 200 mM NaCl + 80 mM EDC) doped with 1× SYBR Gold at *t* = 0 and after 24 h of reaction. Scale bars, 20 μm. Insets show zoomed images of the white squared areas. Scale bars, 10 μm. **c** Schematic representation of the transformation of ISO coacervate droplets into multiphase LC-in-ISO coacervate droplets during ligation. **d** Optical microscopy images of single DDp/*trans*-azoTAB ISO coacervate droplets prepared under reactive conditions (200 mM NaCl + 80 mM EDC) and exposed to cycles of UV and blue light (same light cycles as used in Fig. 3c, i.e., 0–4 h: dark, 4–8 h: UV, 8–24h: blue, 24–28 h: UV, 28–32 h: blue, 32–48 h: UV). Black arrows point to LC subdomains. Scale bars, 5 µm. **e**, **f** Optical microscopy images of single droplets before and after UV (**e**, *t* = 24 h) or blue (**f**, *t* = 28 h) light irradiation during the light cycles shown in (**d**). The dotted line identifies the LC (blue) and ISO (green) phases. Blue arrows point to the LC subdomains. Scale bars, 5 µm. **g** Schematic representation of the selective dissolution of the outer ISO coacervate phase with UV light in multiphase LC-in-ISO coacervate droplets. The remaining LC subphase containing longer polynucleotides acts as a seed for blue light-induced regrowth of the outer ISO coacervate phase.

Samples kept in the dark were further observed before and after a 24-h ligation reaction. Optical microscopy images revealed that soft solids and LC coacervate droplets evolved toward crystalline-like assemblies after the reaction (Fig. 4a), which we attributed to the formation of long covalent polynucleotides that produced less fluid, more ordered complexes^36^. More surprisingly, ISO coacervates exhibited a multiphase droplet organization after ligation, as revealed by the presence of a newly formed birefringent coacervate subphase (Fig. 4a). Droplets doped with the DNA staining dye SYBR Gold further showed a brighter fluorescence within this phase compared to the surrounding coacervate layer (Fig. 4b and Supplementary Fig. 21), suggesting that the inner phase contained a higher nucleotide density. Time-dependent experiments pointed to the gradual growth of this coacervate subphase over time (Supplementary Fig. 22), which correlated with the gradual advancement of the ligation reaction. After 96 h, most of the droplets became completely birefringent and less spherical, with typical textures of columnar LC. Similar reaction-induced phase transitions were observed for sticky ends CG-terminated duplexes, with the formation of LC-in-ISO multiphase droplets (Supplementary Fig. 18). In comparison, control experiments using non-reactive DD oligonucleotides (Supplementary Fig. 22) or non-stacking TT-terminated reactive duplexes (Supplementary Fig. 17) did not undergo structural change over time.

These observations suggested that the coexistence of ISO and LC phases in coacervate droplets was driven by the gradual enrichment of the DNA population in long polynucleotides produced by DDp or 12-CG ligation and their preferential partitioning in the columnar subphase (Fig. 4c). Such behavior is consistent with the higher degree of order expected for long polynucleotides chains compared to shorter ones^36,37,48^ and with spontaneous phase separations predicted and observed in highly polydisperse LC forming DNA solutions^61,62^. This hypothesis was corroborated by the observation that boundaries in the phase diagram of *trans*-azoTAB and the self-complementary 24-mer oligonucleotide DD_2_ (in which the DD sequence was repeated twice: 5’-CGCGAATTCGCGCGCGAATTCGCG-3’) were shifted to higher ionic strength compared to DD (Fig. 1d), so that LC coacervates rather than ISO droplets were produced at 350 mM NaCl for DD_2_/*trans*-azoTAB, and a higher critical coacervation salt concentration was observed compared to DD/*trans*-azoTAB. Length-dependent phase transitions are peculiar to the LC nature of this coacervate system since longer DNA duplexes order at lower concentration^54^, while the increase in coacervate stability with polymer length is well established^45^ and has been observed in oligonucleotide coacervates^43,46^. In addition, multiphase coacervation has been shown to occur when two coacervate systems with sufficiently different critical coacervation salt concentrations are mixed together at lower ionic strength, a condition under which droplets with a sufficient difference in density and surface tension are formed^12,13^. Accordingly, mixtures of DD, DD_2_, and *trans*-azoTAB produced multiphase LC-in-ISO coacervate droplets containing birefringent columnar subdomains at 350 mM NaCl (Supplementary Fig. 23), a condition at which DD alone formed uniformly isotropic coacervate droplets. The fact that LC subdomains are prevalently fully engulfed in the ISO coacervate phase indicates that the surface tension of the inner LC phase (γ_LC_) is higher than the surface tension of the outer ISO phase (γ_ISO_) and than the surface tension between the two phases (γ_ISO-LC_). This in turn can be understood on the basis of the density difference between the interfacing phases ρ_LC_ > ρ_ISO_ ≫ ρ_S_, corresponding to the density of LC droplets, ISO droplets, and supernatant, respectively.

We last undertook microscopy observations of ISO coacervates subjected to cycles of UV and blue light (Fig. 4d). We observed that the coacervate subdomains produced after 24 h were smaller than those formed in samples kept for 24 h in the dark, as expected from the lower ligation yield during light cycling. Notably, when the multiphase droplets were re-exposed to UV light, only the outer ISO phase was disassembled, while the LC coacervate subdomains no longer dissolved (Fig. 4e and Supplementary Movie 3). We attributed this lack of dissolution to the increased stability of phases produced with longer polynucleotides, as suggested by the partial phase diagram of *trans*-azoTAB and DD_2_ compared to DD (Fig. 1d). These undissolved domains subsequently acted as seeds to localize the regrowth of the outer ISO coacervate phase when samples were exposed to blue light (Fig. 4f and Supplementary Movie 4). These results open promising perspectives for selecting the most stable subphases and for reshuffling ligation substrates around coacervates seeds during light cycles (Fig. 4g). The survival of the LC core could also allow to establish compartment lineages if compartment filiation could be performed, which would open new possibilities far beyond transient compartmentalization.

## Discussion

In conclusion, we show that charge-balanced mixtures of an end-to-end stacking oligonucleotide, the Drew–Dickerson dodecamer, and an amphiphilic cationic azobenzene photoswitch produce a rich ensemble of phase-separated complexes, including soft solids, LC and ISO coacervate droplets, depending on oligonucleotide length, azobenzene photo-isomerization, and ionic strength. These chemically enriched phases provide a favorable environment for the non-enzymatic ligation of end-reactive oligonucleotides via carbodiimide chemistry so that long polynucleotides are produced in situ, while the condensation reaction is only poorly effective in the dilute supernatant or in the absence of coacervates. Significantly, polymerization yields are strongly correlated with the nature of the phase in which it occurs, with a markedly increased reaction yield in LC coacervates compared to soft solids or isotropic droplets, indicating that a combination of fluidity and order, typical of liquid crystals, is critical for DD oligonucleotide ligation. Reversible modulation of the assembly and dissolution of coacervate droplets based on light-switchable azobenzene isomerization provides temporal modulation over the ligation reaction in periodically changing environmental conditions, which, if extended to other sequences, could open perspectives for the sequential incorporation of different oligonucleotides along the synthesized polynucleotide chain as a first step toward the emergence of sequence-defined DNA strands. Remarkably, the phase-dependent growth of the DNA population toward longer polymers during DD ligation induces structural changes to the compartments themselves, such as the onset of multiphase organization and gradual ISO-to-LC coacervate transition. Such a correlation between compartment structure and in situ reactions offers a promising platform for compartment-content coupling in protocells. Since coacervates composed of longer polymers are more stable than those made of shorter ones^45^ and LC coacervates are more stable than ISO ones upon an increase in salt and temperature^41,42^, positive feedback may also arise, where oligomer elongation stabilizes the compartment, in turn enhancing elongation. The enhanced ligation and phase transitions reported here strongly depend on the capacity of the model DD sequence to form LC phases. In particular, TT-terminated DD duplexes that are unable to undergo end-to-end stacking due to the non-pairing dangling ends show poor ligation efficiency in coacervates. In comparison, CG-terminated duplexes, in which linear aggregation is favored by base pairing, exhibit similar behavior as DD duplexes. Since many other oligonucleotides with structures compatible with end-to-end linear aggregation, including RNA, replicate the DD phase behavior^36–39^, it is likely that our results can be extended to other sequences.

Overall, this work highlights an unexplored approach to the restructuring of coacervate droplets via in situ enzyme-free oligonucleotide elongation and reveals a strong connection between nucleic acid content and compartment morphology. If generalized to populations of different DNA sequences, these results could be expanded to the emergence of genotype-phenotype coupling in membrane-free protocells, where different DNA sequences (seen as genotype variations) would produce coacervates with different materials properties (seen as a rudimentary form of phenotype differentiation). Combined with self-replication strategies^63^ and recently reported non-equilibrium coacervate growth-division processes^64^, such a system could also open perspectives for a systems chemistry realization of Darwinian evolution in protocell populations with synergistic compartment-content reproduction mechanisms^60^.

## Methods

### Materials

Oligonucleotides were purchased from Integrated DNA Technologies as HPLC-purified, freeze-dried, annealed double-stranded oligomers (sequences are given in Supplementary Table 1); sodium chloride (NaCl) and water-soluble 1-ethyl-3-(3-dimethylaminopropyl) carbodiimide hydrochloride (EDC) were purchased from Sigma-Aldrich; SYBR Gold nucleic acid stain was purchased from ThermoFisher Scientific; azoTAB was synthesized using a three-step reaction by azocoupling *p*-ethoxyaniline with phenol, followed by alkylation with dibromoethane then quaternization with trimethylamine (see Supplementary Methods); denaturing polyacrylamide gels were prepared using gel stock solution from Roth; gel running buffer, 1× Tris-Borate Ethylenediaminetetraacetic acid (TBE) buffer, was prepared from 10× TBE from Roth.

Printed circuit board-mounted LEDs operating at 365 ± 4.5 nm (model M365D2) or 450 ± 9 nm (model M450D3) were purchased from Thorlabs, Inc., and adapted on a custom-made aluminum heat sink. The LEDs were controlled by a T-Cube LED driver that was purchased from Thorlabs, Inc.

Glass coverslips were functionalized with poly(ethylene glycol) brushes to avoid wetting of coacervate droplets. PEGylation was performed by incubation of ethanol-rinsed glass coverslips (0.13–0.17 mm thick) for 48 h into 10 mL toluene solution containing 500 μL of 3-[methoxy(polyethyleneoxy)propyl]tri-methoxysilane (90%, 6–9 PE units, abcr GmbH). Coverslips were subsequently rinsed with ethanol and water and dried with compressed air before being assembled into an observation capillary chamber (as shown in Supplementary Fig. 24).

### Preparation of stock solutions

Stock solutions of double-stranded oligonucleotides were prepared at 25 mM (nucleobase concentration) by dissolving an appropriate amount of the freeze-dried oligonucleotide powder in nuclease-free Milli-Q water in an Eppendorf tube and stored at 4 °C. A 25 mM *trans*-azoTAB aqueous stock solution was prepared by dissolving an appropriate amount of the synthesized azoTAB powder in Milli-Q water in an Eppendorf tube, and the pH adjusted to 8 with NaOH (0.1 M) or HCl (0.1 M). The stock solution was stored in the dark at room temperature before use to ensure complete isomerization to the *trans* state, and then protected from light during use by wrapping the tube with aluminum foil. Sodium chloride (NaCl) stock solutions were prepared at various concentrations (250 mM, 500 mM, 1 M, 2 M, 4 M), and fresh EDC stock solutions were prepared at 400 mM and 1 M concentrations just before use.

### Equilibrium phase behavior studies

Complexes between DD and *trans*-azoTAB were prepared by mixing in the following order aliquots of aqueous stock solutions of *trans*-azoTAB (25 mM, pH 8), NaCl (250 mM–4 M), DD (25 mM nucleobase concentration) to reach the final concentrations of 5 mM *trans*-azoTAB, 5 mM oligonucleotide (nucleobase concentration), and 0–800 mM NaCl. For studies at varying *trans*:*cis*-azoTAB molar ratios, we first mixed a UV-equilibrated *cis*-azoTAB stock solution (25 mM, kept under UV light for at least 30 min) and a dark-adapted *trans*-azoTAB stock solution (25 mM) to produce a mixed *trans*:*cis*-azoTAB stock solution (25 mM, pH 8) at the desired isomer ratio. This new stock solution was used to prepare samples containing different *trans*:*cis*-azoTAB molar ratios. In this case, observations were performed rapidly (<30 min) to limit the spontaneous thermal relaxation of *cis*-azoTAB to *trans*-azoTAB in the dark (half-time of ~20 h^15^) and make sure that the *trans*:*cis*-azoTAB molar ratio did not change during our observations.

Samples were imaged ~15 min after formation by loading an aliquot of the suspension onto a custom-made PEG-functionalized capillary chamber. The complexes (aggregates or droplets) were left to settle for ~5 min on the glass coverslip before imaging. Optical microscopy imaging was performed on an inverted Leica DMI 4000B microscope using a ×63 oil immersion objective (HCX PL APO, 1.4 NA), images acquired using MicroManager (v1.4), and on an inverted Nikon Ti-U polarized microscope using ×20 or ×50 objectives, images acquired using Nikon NIS Element AR (v5.30.03). UV or blue-light irradiation was performed in situ on the optical microscope by using a CoolLED light source operating at 365 nm (UV) or 450 nm (blue) or a Nikon epifluorescence mercury lamp (OSRAM HBO 103W/2) and Nikon DAPI and FITC filter cubes for UV and blue light, respectively. Images were processed using FIJI (ImageJ) software (v1.53f).

Turbidity measurements were also used to characterize the salt- and light-dependent phase behavior of DD and azoTAB. Here, freshly made samples were added to a 384-well plate and incubated for ~5 min, then the absorbance at 700 nm was measured on a Molecular Device SpectraMax Paradigm microplate reader using SoftMax Pro software (v6.2.2), and a reference value (corresponding to 25 μL of pure water) was subtracted. Cycles of UV and blue light were performed by irradiating the samples for 15 min using alternating UV and blue LED (Thorlabs, Inc): the samples were irradiated for 15 min under UV light using an LED operating at 365 nm, then the absorbance was measured and the samples were irradiated for 15 min under blue light with an LED operating at 450 nm, and the turbidity monitored at 700 nm after each irradiation.

### Fraction of oligonucleotides in dense complexes vs. supernatant

The fraction of DD sequestered in LC and ISO coacervates vs. supernatant was measured by UV-vis spectroscopy after the separation of azoTAB and DD by size exclusion chromatography. Typically, 30 µL of a suspension of *trans*-azoTAB/DD coacervate droplets was produced in an Eppendorf tube (5 mM nucleobase concentration, 5 mM *trans*-azoTAB, 200 or 350 mM NaCl for LC or ISO coacervates, respectively), and separated in three 10-μL aliquots. One aliquot was equilibrated in the dark (10 min), another one was irradiated with UV light (10 min), and the last one was first exposed to UV light (10 min) followed by blue-light irradiation (10 min). Each sample was then centrifuged (10 min, 10,600 × *g*), and the supernatant was collected, supplied with 2 μL of 5 M NaCl, and further irradiated with UV light to induce complete dissociation of any potential DD/azoTAB complexes. Then, 10 µL of 1 M NaCl were added to the initial tubes after supernatant removal (we observed that these tubes contained a sedimented bulk coacervate phase for dark and blue light conditions), and the samples were further irradiated under UV light to induce complete dissociation of DD and *cis*-azoTAB. All samples were added to a PD10 desalting column (GE Healthcare) equilibrated with Milli-Q water to separate azoTAB and DD. Fractions containing only DD were collected and the concentration of oligonucleotide was determined in each of them by UV-vis spectroscopy at 260 nm, using an extinction coefficient of 189,089 L mol^−^^1^ cm^−^^1^ for double-stranded DD^65^. The total amount of DD in bulk coacervate phases and supernatant were determined from the concentration of DD in each fraction and the volume of the fractions (measured by pipetting).

### Non-enzymatic oligonucleotide ligation conditions

Time-dependent oligonucleotide ligation experiments in the dark were performed in 0.2-mL Eppendorf tubes containing 10 µL of reaction solutions: 5 mM *trans*-azoTAB, 0.42 mM 3’-phosphate DNA 12-mer, DDp (corresponding to 5 mM negative charge concentration), 80 mM EDC, and 0, 100, or 200 or 400 mM NaCl for soft solids, LC, and ISO coacervates formation or single phase, respectively. Before PAGE analysis, samples were centrifuged for 10 min at 5,000 × *g* to ensure dense phase sedimentation, then 9 µL of supernatant was extracted by pipetting and transferred to 11 µL stop solution (composed of 1 µL of 4 M NaCl and 10 µL of 50 mM ethanolamine in water), while the remaining 1 µL at the bottom of the tube was diluted with 11 µL of stop solution. The same protocol was followed to perform ligation reaction for DD-TTp and 12-CGp oligonucleotides, except that: (1) the initial DD-TTp concentration corresponding to 5 mM negative charge concentration was 0.36 mM; (2) 20, 80, 160 mM EDC were tested; (3) for 12-CGp soft solids, LC, and ISO coacervates formation were obtained at 50, 150, and 250 mM NaCl, respectively. For different reaction times, samples prepared from the same initial solution were incubated in different tubes and stopped at decided time intervals. For reactions performed under varying light irradiation conditions (continuous dark, continuous UV, or UV/blue cycles), the 10 μL reactive solutions were incubated in non-binding treated plastic surface 384-well plates (Corning 3544). To prevent evaporation in the well plates, samples were covered with 20 µL of mineral oil light (Sigma-Aldrich) and plates were sealed with adhesive PCR plate film (Biorad). UV or blue-light irradiation was performed in situ on a Nikon Ti-U inverted optical microscope equipped with a ×2 objective, allowing the illumination of the full well area, by using a Nikon epifluorescence mercury lamp (OSRAM HBO 103W/2) and Nikon DAPI and FITC bandpass filter cubes for UV and blue light (10 s exposure every 2 min), respectively. These light sources are far enough from the UV adsorption peak of DNA (260 nm) so that no degradation is expected. Reactions were stopped by the addition of 2 μL 4 M NaCl to cause coacervates disassembly and 20 μL of 50 mM ethanolamine solution. For different reaction times, samples prepared from the same initial solution were incubated in different wells and stopped at decided time intervals.

### Polyacrylamide gel electrophoresis

Ligation products analysis was performed using 15% denaturing polyacrylamide gels (20 cm wide × 16 cm high) containing 8.3 M urea and run in 1× TBE for 3 h at a constant power of 32 W. Equal DNA masses from each sample, 0.5 μg, were loaded in the gels. Gels were stained with 1× SYBR Gold for 20 min with gentle agitation. Ladders used in gels were composed of an equal mass mixture of 12-, 24-, 48-, and 96-mer DNA sequences, corresponding to 1-, 2-, 4-, and 8-times repetitions of the DD sequence, respectively, unless otherwise specified. Uncropped gels are shown in a Source Data file.

Gel image analysis was carried out with FIJI (ImageJ) software (v1.53f) using a custom MATLAB (MathWorks) script. Estimations of the ligation product distributions from gel band intensities were performed by measuring the profile peaks areas normalized on the sum of the area of the peaks. Calculations of reaction yield were obtained by fitting the data with the cumulative product distribution obtained from Flory’s theory for simple polymerization^57^ (see Supplementary Notes 2 and 3) using OriginLab 2019 (v 9.6.0.172).

### Sample imaging during ligation

Reactive samples were prepared by mixing 5 mM *trans*-azoTAB, 0.42 mM 3’-phosphate DNA 12-mer DDp (corresponding to 5 mM negative charge concentration) supplemented with 1× SYBR Gold, 80 mM EDC, and 0, 100, or 200 mM NaCl for soft solids, LC, and ISO coacervates formation, respectively. Samples were loaded in a custom-made capillary chamber (Supplementary Fig. 24) that was sealed with UV-curing glue (while protecting the samples from UV light exposure using foil). No evaporation was observed in the sealed chamber for up to 2 weeks. Samples were imaged at different times during the reaction on an inverted Leica DMI 4000B microscope using a ×63 oil immersion objective (HCX PL APO, 1.4 NA). Light cycling during ligation was performed using the in situ-mounted CoolLED and appropriate bandpass filters to select the irradiation wavelength (UV, 365 nm; blue, 450 nm). Confocal fluorescence microscopy imaging was performed on an inverted Leica DMI RE2 scanning laser confocal microscope. DD-TTp and 12-CGp samples were prepared following the same protocol but without SYBR Gold addition and imaged on an inverted Nikon Ti-E microscope using a ×60 objective (CFI S Plan Fluor ELWD ADL 60XC, 0.7 NA).

### Fluorescence recovery after photobleaching

Fluorescence recovery after photobleaching (FRAP) experiments were performed on a Leica DMI8 scanning laser confocal microscope equipped with a ×25 water immersion objective (HC FLUOTAR L, 0.95 NA). Coacervate samples were prepared by doping DD solutions with 1:1000 Cy5-labeled DD oligomers (Integrated DNA Technologies). Images were analyzed by the Leica LAS X software (v 3.5.7.23225). The normalized FRAP intensity I_FRAP_(t) was calculated as [S(t) − B(t)]/[C(t) − B(t)], where S(t), C(t), and B(t) are the fluorescence intensity measured in circular ROIs with the same diameter (2–5 μm) inside the bleached coacervate area (sample, S), non-bleached coacervate region (control, C), and non-bleached supernatant (background, B). I_FRAP_(t) was fitted with a monoexponential growth function A.exp(−t/τ) to extract the fluorescent recovery time constant, τ.

### Statistics and reproducibility

Quantitative comparisons of ligation reactions were performed on samples from the same experiment and derived from analysis of lanes belonging to the same gel or gels run in parallel when this was not possible. Gels replicas on two independent experiments were performed for all experiments to qualitatively confirm reproducibility. All shown micrographs are representative images of two different fields of view of two independent experiments.

### Reporting summary

Further information on research design is available in the Nature Portfolio Reporting Summary linked to this article.

## Supplementary information

Supplementaty Information

Description of Additional Supplementary Files

Supplementary Movie 1

Supplementary Movie 2

Supplementary Movie 3

Supplementary Movie 4

Reporting Summary

## Data Availability

The data supporting the findings of this study are available within the article and its Supplementary Information. Numeric data underlying all plots and gels in the main publication are available as Source Data files. All other data are available from the corresponding author upon request. Source data are provided with this paper.

## Source data

Source Data

## Code availability

The MATLAB code used to analyze the gels can be found at https://github.com/tfraccia/gel_lane_analysis-code.
