# Supplementary material for: Non-enzymatic oligonucleotide ligation in coacervate protocells sustains compartment-content coupling
Source: Nat Commun. 2023 May 9;14:2606. doi: 10.1038/s41467-023-38163-8 (PMC10169843; doi:10.1038/s41467-023-38163-8)
Supplement: Supplementary file 1 — Supplementaty Information [file 41467_2023_38163_MOESM1_ESM.pdf]

Supplementary Information

Non-enzymatic oligonucleotide ligation in coacervate protocells sustains compartment-content coupling

Tommaso P. Fraccia & Nicolas Martin

Supplementary Methods **2**
Supplementary Table 1 | Oligonucleotide sequences 2
Synthesis of *trans*-azoTAB 3

Supplementary Figures **4**
Supplementary Fig. 1 | Phase behaviour of DD and *trans*-azoTAB with salt 4
Supplementary Fig. 2 | Liquid-like behaviour of coacervate micro-droplets 4
Supplementary Fig. 3 | Images of LC coacervate droplets 5
Supplementary Fig. 4 | Phase behaviour of non-stacking oligonucleotides 5
Supplementary Fig. 5 | FRAP in DD/*trans*-azoTAB complexes 6
Supplementary Fig. 6 | Evidence of hydrophobic interactions for DD/*trans*-azoTAB complexation 7
Supplementary Fig. 7 | Oligonucleotide/azoTAB equilibrium phase separation behaviour 8
Supplementary Fig. 8 | Photoswitchable behaviour of azoTAB 9
Supplementary Fig. 9 | Photoswitchable behaviour and LC coacervate microdroplets vs. salt-free solid-like aggregates 10
Supplementary Fig. 10 | Reversibility of the light-driven droplet dissolution/condensation process 11
Supplementary Fig. 11 | Scheme of the chemical ligation of end-reactive DDp oligonucleotides 11
Supplementary Fig. 12 | Influence of EDC on oligonucleotide/azoTAB phase separation 12
Supplementary Fig. 13 | Time-dependent full polyacrylamide electrophoresis gels 13
Supplementary Fig. 14 | Influence of EDC concentrations on ligation 14
Supplementary Fig. 15 | Average degree of polymerization during oligonucleotide ligation 15
Supplementary Fig. 16 | Product distribution during oligonucleotide ligation 16
Supplementary Fig. 17 | Ligation experiments with DD-TTp duplexes 17
Supplementary Fig. 18 | Ligation experiments with 12-CGp duplexes 18
Supplementary Fig. 19 | Polyacrylamide gel electrophoresis for dark and UV/blue cycles 19
Supplementary Fig. 20 | Results of DDp ligation as a function of time for reactions performed in the dark, under UV or using UV/blue cycles 20
Supplementary Fig. 21 | Oligonucleotide elongation produces multiphase coacervates 20
Supplementary Fig. 22 | Imaging of ISO coacervates over reaction time 21
Supplementary Fig. 23 | Mixed oligonucleotide lengths form multiphase LC-in-ISO coacervate droplets with *trans*-azoTAB 22
Supplementary Fig. 24 | Schematic view of the custom-made capillary chamber used to image coacervate droplets 23
Supplementary Fig. 25 | Plots of the linear condensation polymers distributions from Flory's theory 23
Supplementary Fig. 26 | Illustrative steps of the analysis of electrophoretic polyacrylamide gels 24

Supplementary Notes **25**
Supplementary Note 1 | Estimation of the oligonucleotide concentration in dark-adapted ISO coacervate droplets 25
Supplementary Note 2 | Flory's theory for linear condensation of polymers 26
Supplementary Note 3 | Analysis of the fluorescence intensity profiles of PAGE runs 27
Supplementary Note 4 | Illustrative steps of the analysis of electrophoretic polyacrylamide gels 28

Supplementary References **29**

Supplementary Methods

Supplementary Table 1. Oligonucleotide sequences.

Name	Sequence (5', 3')	Length (nt)	Molecular weight ($g.mol^{-1}$)
DD	CGCGAATTCGCG	12	3 646.4
DDp	CGCGAATTCGCG - phosphate	12	3 726.4
DD-TT	CGCGAATTCGCGTT	14	4 253.8
DD-TTp	CGCGAATTCGCGTT - phosphate	14	4 334.8
12-CGp	GCGTTAACGCCG - phosphate	12	3 726.4
20 bp	CGCGCGAAAATTTTCGCGCG	20	6 118.0
DD_2	CGCGAATTCGCGCGCGAATTCGCG	24	7 292.8
DD_4	CGCGAATTCGCGCGCGAATTCGCGCGCGAA TTCGCGCGCGAATTCGCG	48	14 585.6
DD_8	CGCGAATTCGCGCGCGAATTCGCGCGCGAA TTCGCGCGCGAATTCGCGCGCGAATTCGCG CGCGAATTCGCGCGCGAATTCGCGCGCGAA TTCGCG	96	29 171.2

Synthesis of trans-azoTAB

The synthesis of trans-azoTAB was adapated rom previously reported protocols (see ref. 1).

Synthesis of 4-ethoxy-4'-hydroxy-azobenzene (azoH): Concentrated HCl (17 mL) and ice (80 g) were added to a 1:1 v/v ethanol : water solution (160 mL) containing p-ethoxyaniline (10.3 mL, 80 mmol, 1 equiv.) and sodium nitrite (5.5 g, 80 mmol, 1 equiv.) in an ice bath (T = 0°C) in a fume hood. The mixture was stirred for 1h. Cold water (42 mL) containing phenol (7.5 g, 80 mmol, 1 equiv.) and NaOH (6.4 g, 160 mmol, 2 equiv.) was then carefully added to the solution and the mixture was stirred for another 90 min by keeping the temperature below 5°C. The pH of the solution was then adjusted to 1 with concentrated HCl and left to stand for 30 min. The resulting precipitate was filtered, thoroughly washed with water and dried under vacuum overnight to give 4-ethoxy-4'-hydroxyazobenzene (azoH) as a dark brown powder (76% yield). ^{1}H NMR (400 MHz, $CDCl_3$): δ = 7.86 (d, ^{3}J(H-H) = 8 Hz, 2H; Ar-H), 7.82 (d, ^{3}J(H-H) = 8 Hz, 2H; Ar-H), 6.99 (d, ^{3}J(H-H) = 8 Hz, 2H; Ar-H), 6.94 (d, ^{3}J(H-H) = 8 Hz, 2H; Ar-H), 4.12 (q, ^{3}J(H-H) = 6 Hz, 2H; CH_2), 1.46 ppm (t, ^{3}J(H-H) = 4 Hz, 3H; CH_3); ^{13}C NMR (400 MHz, $CDCl_3$): δ = 161.0 (Ar-C), 157.9 (Ar-C), 147.1 (Ar-C), 146.8 (Ar-C), 124.6 (Ar-C), 124.4 (Ar-C), 115.8 (Ar-C), 114.7 (Ar-C), 63.8 (CH_2O), 14.8 ppm (CH_3).

Synthesis of 4-ethoxy-(4'-(2-bromoethyloxy)phenyl)azobenzene (azoBr): A mixture of 4-ethoxy-4'-hydroxy-azobenzene (2.4 g, 10 mmol, 1 equiv.), 1,2-dibromoethane (5.6 g, 3 equiv.), potassium carbonate (2.07 g, 1.5 equiv.) and potassium iodide (0.083 g, 0.05 equiv.) were refluxed in 50 mL of butanone for 48 h in the dark in a fume hood. The reaction mixture was filtered hot to remove solid impurities and salt, and the residue was washed with butanone. The filtrate was collected and the solvent was removed under reduced pressure. The obtained solid was dissolved in dichloromethane (20 mL) and extractions were performed with NaOH solution (1M, 2 × 8 mL) then pure water (2 × 8 mL). The organic phase was dried with $MgSO_4$ and concentrated. The crude product was recrystallized with hot filtration from ethanol and dried under vacuum to give 4-ethoxy-(4'-(2-bromoethyloxy)phenyl)azobenzene (azoBr) as an orange powder (54% yield). ^{1}H NMR (400 MHz, $CDCl_3$): δ = 7.92 (q, ^{3}J(H-H) = 8.2 Hz, 4H; Ar-H), 7.00 (dd, ^{3}J(H-H) = 8.6 Hz, 4H; Ar-H), 4.37 (t, ^{3}J(H-H) = 8 Hz, 2H; CH_2O), 4.12 (q, ^{3}J(H-H) = 6 Hz, 2H; CH_2O), 3.67 (t, ^{3}J(H-H) = 7 Hz, 2H; CH_2Br), 1.46 ppm (t, ^{3}J(H-H) = 6 Hz, 3H; CH_3); ^{13}C NMR (400 MHz, $CDCl_3$): δ = 161.5 (Ar-C), 160.2 (Ar-C), 146.8 (Ar-C), 146.3 (Ar-C), 124.8 (Ar-C), 124.6 (Ar-C), 114.9 (Ar-C), 114.8 (Ar-C), 68.0 (CH_2O), 63.9 (CH_2O), 28.8 (CH_2Br), 14.8 ppm (CH_3).

Synthesis of azobenzene trimethylammonium bromide (azoTAB): 1 g of 4-ethoxy-(4'-(2-bromoethyloxy)phenyl)azobenzene (4.4 mmol, 1 equiv.) was dissolved in 80 mL of dry THF, followed by the addition of a 33% solution of trimethylamine in ethanol (4.2 mL, 11.5 mmol, 4 equiv.). The mixture was stirred for 6 days in the dark in a fume hood. The resulting precipitate was filtered, washed with THF, and dried under vacuum. The crude product was recrystallized twice from ethanol and dried under vacuum overnight to give azobenzene trimethylammonium bromide (azoTAB) as an orange powder (36% yield). ^{1}H NMR (400 MHz, DMSO): δ = 7.86 (d, ^{3}J(H-H) = 8 Hz, 2H; Ar-H), 7.82 (d, ^{3}J(H-H) = 8 Hz, 2H; Ar-H), 7.17 (d, ^{3}J(H-H) = 8 Hz, 2H; Ar-H), 7.08 (d, ^{3}J(H-H) = 8 Hz, 2H; Ar-H), 4.56 (m, 2H; CH_2O), 4.11 (q, ^{3}J(H-H) = 6 Hz, 2H; CH_2O), 3.82 (m, 2H; CH_2N), 3.18 (s, 9H; CH_3N), 1.35 ppm (t, ^{3}J(H-H) = 4 Hz, 3H; CH_3); ^{13}C NMR (400 MHz, DMSO): δ = 161.3 (Ar-C), 159.9 (Ar-C), 147.1 (Ar-C), 146.4 (Ar-C), 124.7 (Ar-C), 124.5 (Ar-C), 115.8 (Ar-C), 115.4 (Ar-C), 64.5 (CH_2O), 64.1 (CH_2N), 62.5 (CH_2O), 53.6 (CH_3N), 15.0 ppm (CH_3).

Supplementary Figures

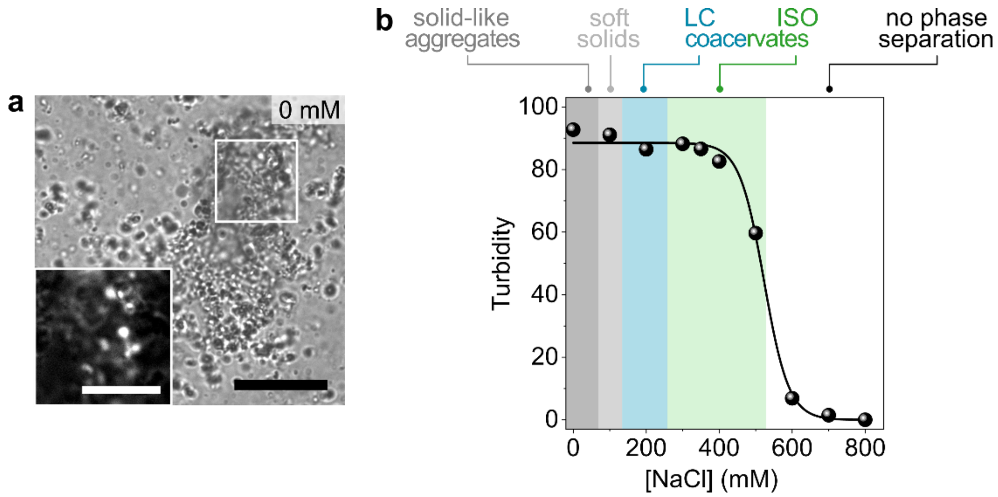

Supplementary Figure 1. Phase behaviour of DD and *trans*-azoTAB with salt. a, Optical microscopy image of an equimolar charge mixture of DD (5 mM nucleobase concentration) and *trans*-azoTAB (5 mM) prepared in pure water (0 mM NaCl), showing the formation of solid-like, amorphous precipitates. Scale bar, 20 µm. Inset shows a zoomed image of the white squared area under 90° crossed polarisers. Scale bar, 10 µm. **b**, Evolution of the turbidity at 700 nm of charge-balanced mixtures of DD (5 mM nucleobase concentration) and *trans*-azoTAB (5 mM) as a function of the NaCl concentration. The existence domains and boundaries of each type of complexes produced were identified by optical microscopy imaging and are shown as coloured areas (dark grey: solid-like aggregates, light grey: soft solids, cyan: liquid crystalline (LC) coacervates, green: isotropic (ISO) coacervates).

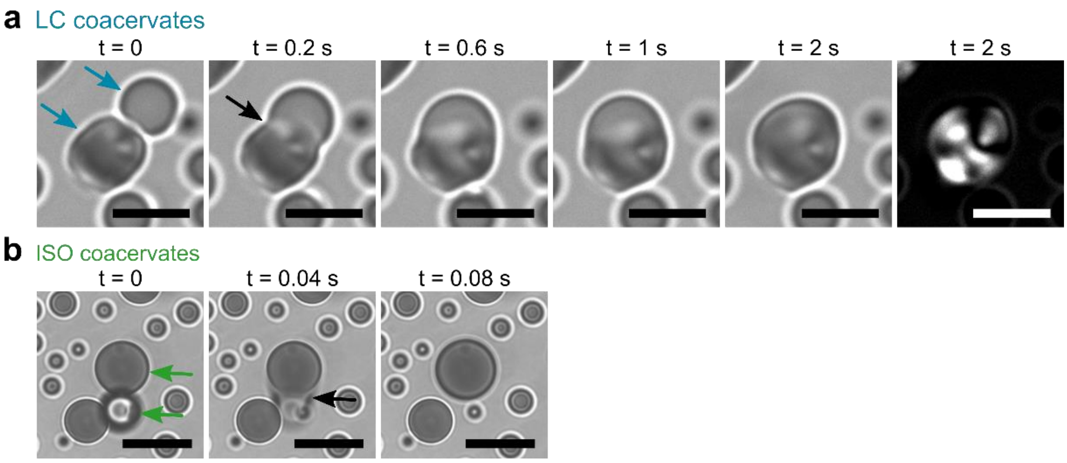

Supplementary Figure 2. Liquid-like behaviour of coacervate micro-droplets. a,**b**, Time series of optical microscopy images of 5 mM equimolar charge ratio DD/*trans*-azoTAB coacervate microdroplets prepared at 200 mM NaCl (**a**, liquid crystalline (LC) droplets) or 350 mM NaCl (**b**, isotropic (ISO) droplets), showing fusion of two contacting droplets (identified with cyan or green arrows, respectively). The last image in **a** was taken after fusion under 90° crossed polarisers. Scale bars, 5 µm. The fusion behaviour supports the liquid-like nature of both LC and ISO coacervates. Note the difference in the timescales: the fusion of LC droplets (**a**) is much slower compared to ISO droplets (**b**) due to LC domains that have to re-arrange during coalescence.

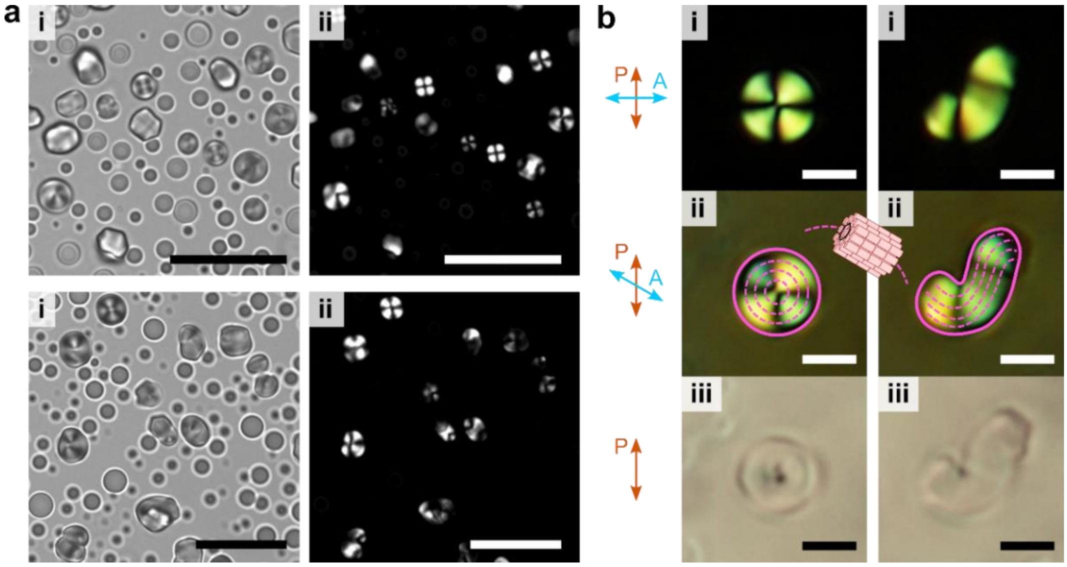

Supplementary Figure 3. Images of LC coacervate droplets. **a**, Optical microscopy images in bright-field (i) and under 90° crossed polarisers (ii) of DD/*trans*-azoTAB LC coacervate microdroplets prepared at equimolar charge ratio (5 mM) in the presence of 200 mM NaCl. Scale bars, 20 µm. Images show many LC droplets in the fields of view that coexist with non-birefringent droplets. **b**, Zoom of single DD/*trans*-azoTAB LC coacervate compartments prepared as in **a** under 90° crossed polarisers (i), partially de-crossed polarisers (ii) and analyser removed (iii). Scale bars, 5 µm. Images in (ii) show birefringent "focal conics" textures typical of columnar *ds*DNA liquid crystals from which it is possible to identify the microscopic arrangement of DD/*trans*-azoTAB complexes, forming hexagonal bundles (sketch) and orienting tangentially to the superimposed magenta dashed lines.

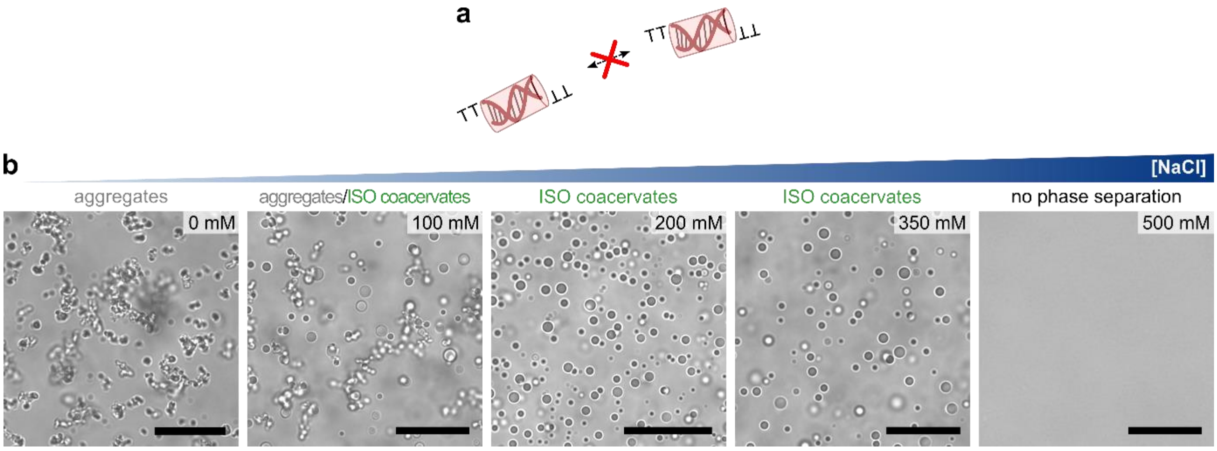

Supplementary Figure 4. Phase behaviour of non-stacking oligonucleotides. **a**, Addition of 3'-TT dangling-ends to DD (5'-CGCGAATTCGCGTT-3', DD-TT) inhibits end-to-end stacking interactions. **b**, Optical microscopy images of DD-TT/*trans*-azoTAB mixtures (5 mM charges) at varying NaCl concentrations. Scale bars, 20 µm. No LC coacervate droplets are observed on the whole salt concentration range tested; rather, solid-like aggregates produced in the absence of salt transform into ISO coacervate droplets when the ionic strength increases. Coexistence of aggregates and ISO droplets is observed at intermediate salt concentrations (100 mM NaCl). Due to the absence of end-to-end interactions, the DNA duplex with TT overhangs is much more flexible and shorter than physically connected DD strands, which favours the formation of unstructured ISO coacervate phases on a broader salt range rather than soft solids or LC droplets.[2]

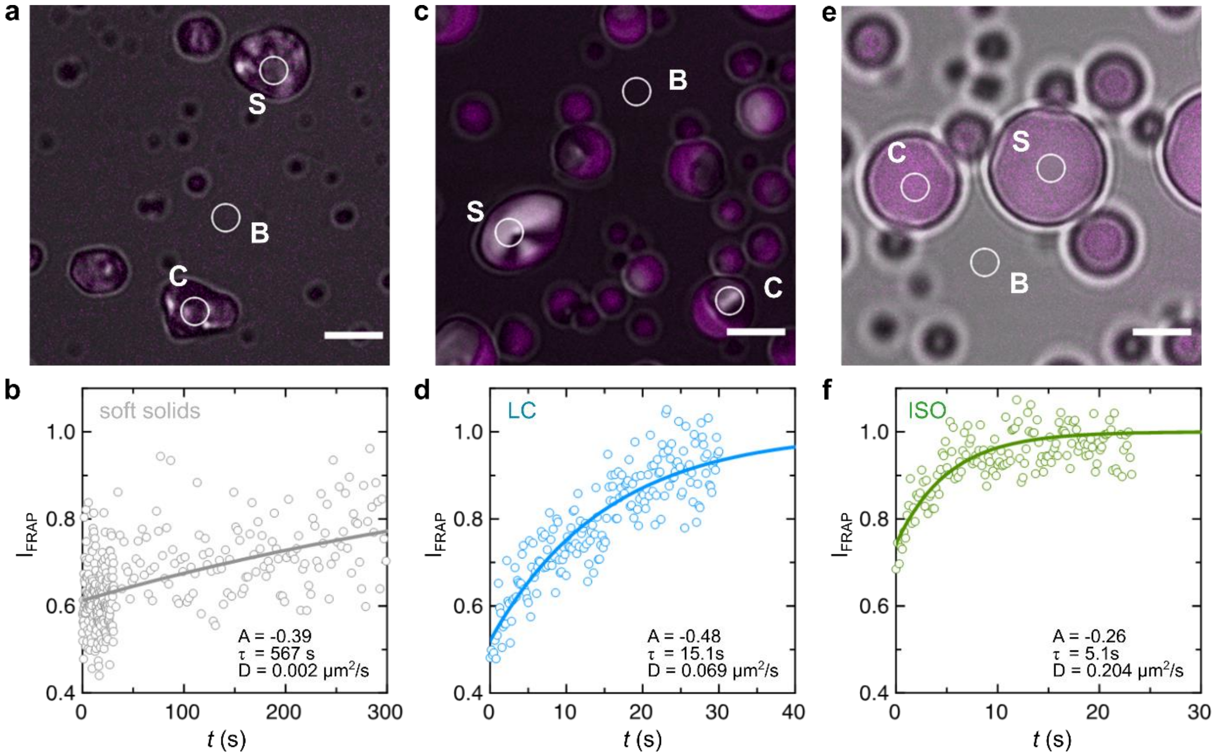

Supplementary Figure 5. FRAP in DD/*trans*-azoTAB complexes. **a**, **c**, **e**, Confocal fluorescence microscopy images of ISO (**a**) or LC (**c**) coacervates and soft solids (**e**) prepared at equimolar charge mixture of DD doped with a 1:1000 fraction of Cy5 labelled DD oligomers (5 mM base concentration) and trans-azoTAB (5 mM) in the presence of 350 mM (ISO), 200 mM (LC) or 100 mM (soft solids) NaCl. The regions labelled C, S and B refer to a non-bleached control area within the complexes, the bleached area and the background control in the supernatant, respectively, used for analysis (see Methods). **b**, **d**, **f**, Normalized fluorescence intensity recovery after photobleaching I_{FRAP} in the samples shown in **a**, **c**, **e**. Data is fitted with a monoexponential growth I_{FRAP} = A.exp(-t/τ) , and the parameters of the fits provided. Results show that fluorescence recovery (hence fluidity) is ca.3-fold and 110-fold faster in ISO droplets compared to LC coacervates and soft solids, respectively.

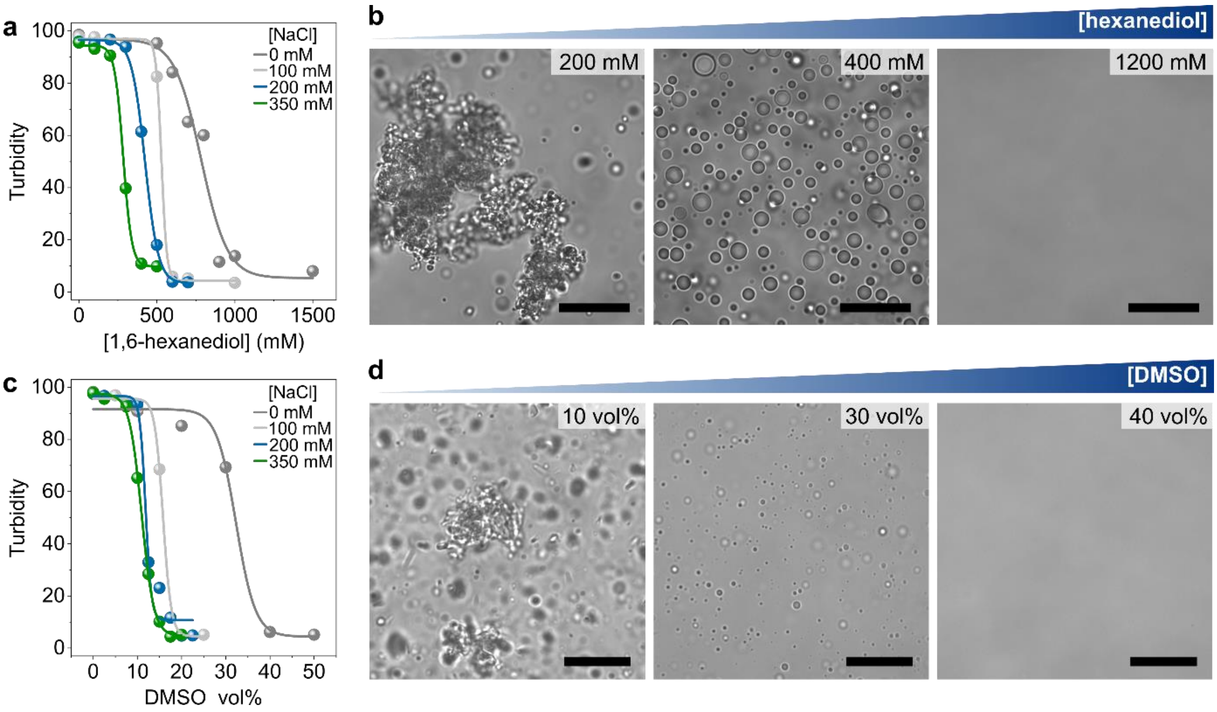

Supplementary Figure 6. Evidence of hydrophobic interactions for DD/*trans*-azoTAB complexation. **a**,**c** Evolution of the turbidity at 700 nm of charge-balanced mixtures of DD (5 mM nucleobase concentration) and *trans*-azoTAB (5 mM) prepared at different ionic strength as a function of 1,6-hexanediol concentration (**a**) or DMSO volume fraction (**c**) used to screen weak hydrophobic interactions. The former is an aliphatic alcohol commonly used to disrupt weak hydrophobic interactions between aromatic residues in membraneless organelles;[3] the latter is a water-miscible organic solvent that solubilises hydrophobic moieties. **b**,**d**, Optical microscopy images of DD/trans-azoTAB complexes prepared at 0 mM NaCl and increasing concentrations of 1,6-hexanediol (**b**) or DMSO (**d**), as indicated. Scale bars, 20 µm. Solid-like aggregates transform into ISO coacervates, then dissolve, pointing to the role of weak hydrophobic interactions in the stabilization of complexes.

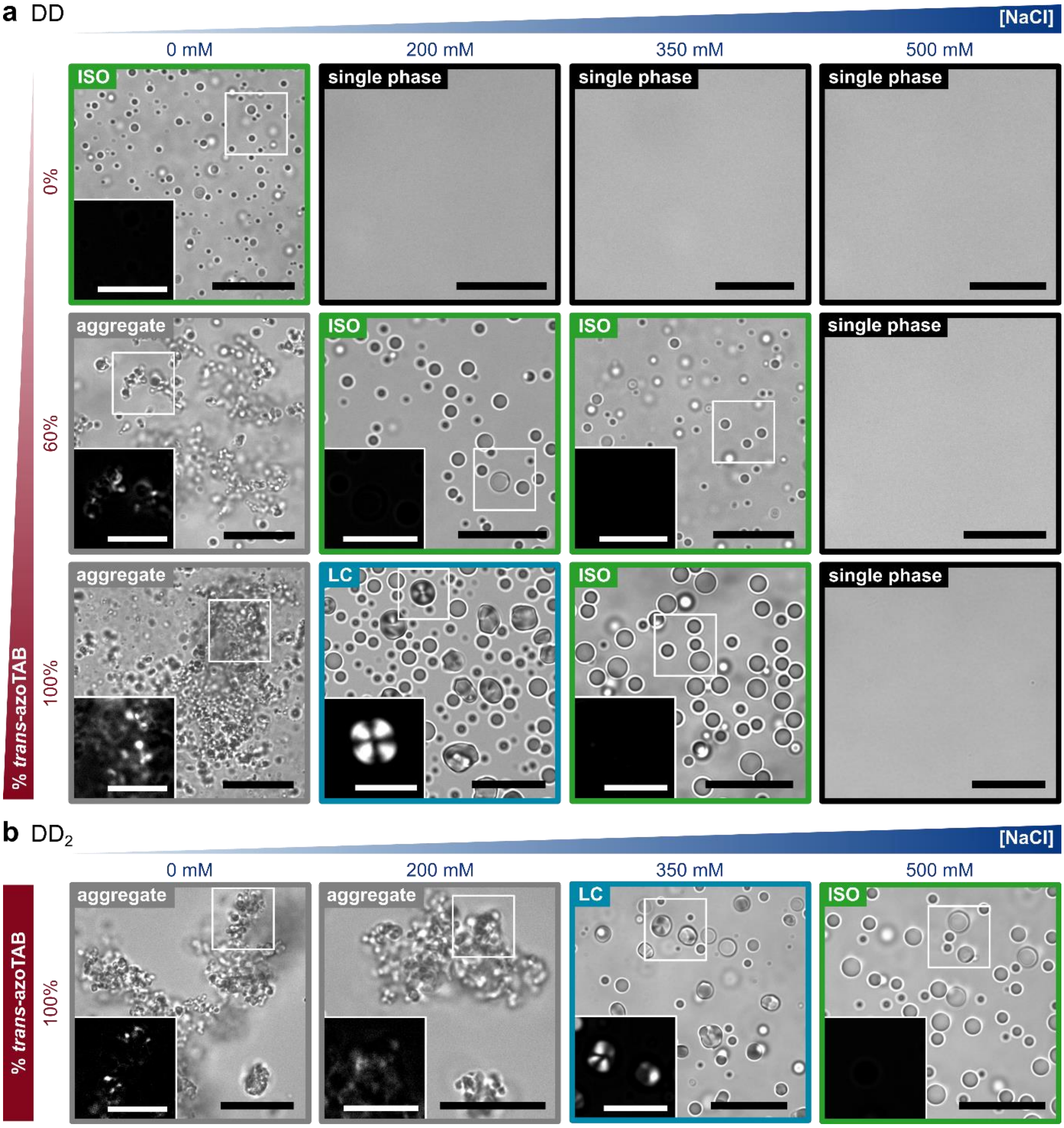

Supplementary Figure 7. Oligonucleotide/azoTAB equilibrium phase separation behaviour. a, Optical microscopy images of DD/azoTAB equimolar charge mixtures prepared at 5 mM total azoTAB concentration but varying *trans*:*cis* composition in the presence of varying NaCl concentrations, as indicated. Scale bars, 20 µm. Insets show zoomed images of the white squared area under crossed polarisers. Scale bars, 10 µm. **b**, Optical microscopy images of DD_2/azoTAB equimolar charge mixtures (5 mM nucleobase concentration, 5 mM *trans*-azoTAB) in the presence of varying NaCl concentrations, as indicated. Scale bars, 20 µm. Insets show zoomed images of the white squared area under crossed polarisers. Scale bars, 10 µm. Such systematic optical microscopy imaging was used to plot the equilibrium phase diagram reported in Fig. 1d.

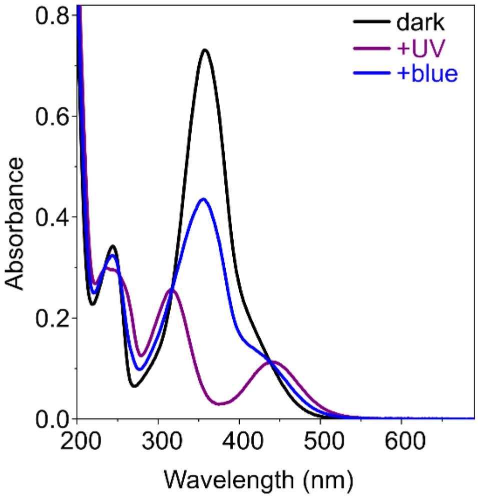

Supplementary Figure 8. Photoswitchable behaviour of azoTAB. UV/vis spectra of a 30 µM solution of pure azoTAB in the dark (black line), under UV light (purple line) or under blue light (blue line). The fractions of *trans*- and *cis*-azoTAB can be estimated from these spectra, as detailed previously:[1] dark: 100% *trans*-azoTAB; UV: 4% *trans*-azoTAB and 96% *cis*-azoTAB; blue: 58% *trans*-azoTAB and 42% *cis*-azoTAB. Note that exposure of UV-adapted *cis*-azoTAB to blue light restores a fraction of *trans*-azoTAB of only 58%, a value significantly lower than in dark-adapted solutions.

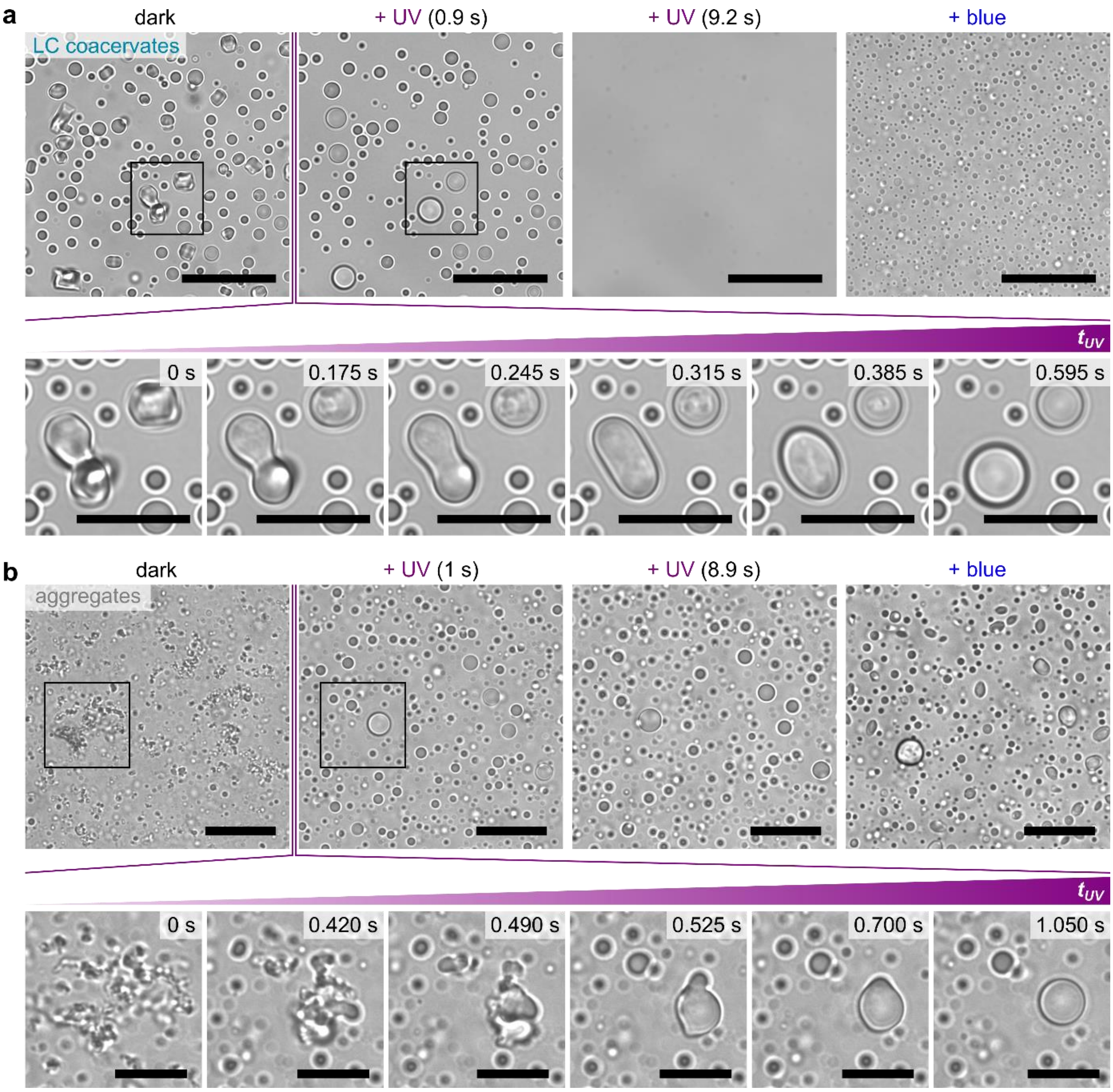

Supplementary Figure 9. Photoswitchable behaviour and LC coacervate microdroplets vs. salt-free solid-like aggregates. **a**, Optical microscopy images of charge-balanced DD/*trans*-azoTAB LC coacervate droplets (10 mM total charge concentration) prepared at 200 mM NaCl in the dark, or exposed to UV or blue light, as indicated. Scale bars, 20 µm. UV-exposed LC droplets first transform to ISO droplets before dissolution, as highlighted in the zoomed images (black square in **a**, scale bars, 10 µm), and reassemble as ISO droplets under blue light, as expected from equilibrium phase diagram studies. **b**, Optical microscopy images of charge-balanced DD/*trans*-azoTAB solid-like aggregates (10 mM total charge concentration) prepared in pure water (no added salt) in the dark, or exposed to UV, as indicated. Scale bars, 20 µm. UV-exposed solid-like aggregates transform to ISO droplets, as highlighted in the zoomed images (black square in **b**, scale bars, 10 µm), but do not dissolve, as expected from equilibrium phase diagram studies, and reassemble as soft solids under blue light, as expected from equilibrium phase diagram studies.

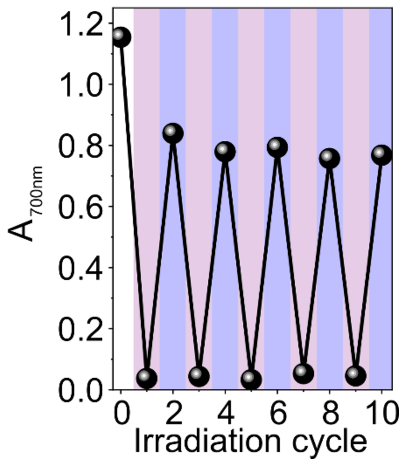

Supplementary Figure 10. Reversibility of the light-driven droplet dissolution/condensation process. Plot of the absorbance at 700 nm of a mixture of DD (5 mM nucleobase concentration) and *trans*-azoTAB (5 mM) prepared at 350 mM NaCl (ISO droplets) after multiples irradiations cycles: 0 = dark, odd numbers = UV light (purple areas), even numbers = blue light (blue areas). Results show that the dissolution and condensation of coacervate droplets can be repeated multiple times.

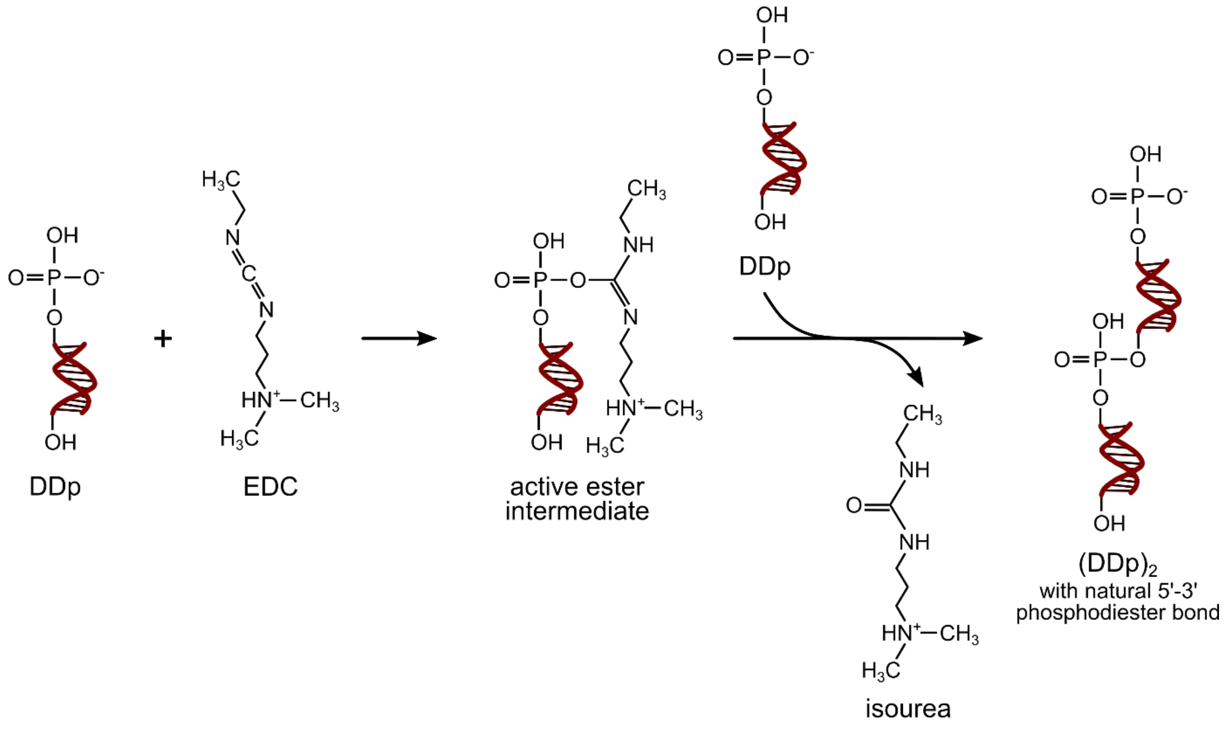

Supplementary Figure 11. Scheme of the chemical ligation of end-reactive DDp oligonucleotides. EDC activates the terminal phosphate groups of DDp to produce reactive ester intermediates able to react with the 5’ hydroxyl terminal groups, thereby creating a native 5’-3’ covalent phosphodiester bond.

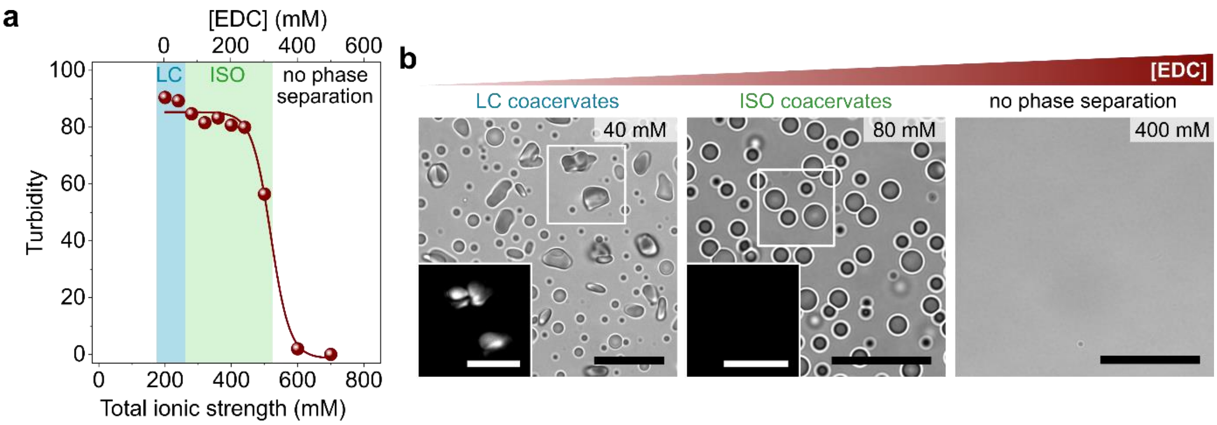

Supplementary Figure 12. Influence of EDC on oligonucleotide/azoTAB phase separation. **a**, Evolution of the turbidity at 700 nm of charge-balanced mixtures of DD (5 mM nucleobase concentration) and *trans*-azoTAB (5 mM) prepared at 200 mM NaCl and increasing EDC concentrations. Coloured areas indicate the nature of the complex produced derived from optical microscopy imaging (cyan: LC coacervates, green: ISO coacervates). A similar evolution as with NaCl only is observed. **b**, Optical microscopy images of DD/*trans*-azoTAB mixtures prepared at 5 mM *trans*-azoTAB concentration and equimolar charge ratio in the presence of 200 mM NaCl and varying EDC concentrations. Scale bars, 20 µm. Insets show zoomed images of the white square area under 90° crossed polarisers. Scale bars, 10 µm. We observed that increasing EDC concentration produced a similar LC-to-ISO coacervate droplets transition as with NaCl only, contributing to the total ionic strength of the solution.

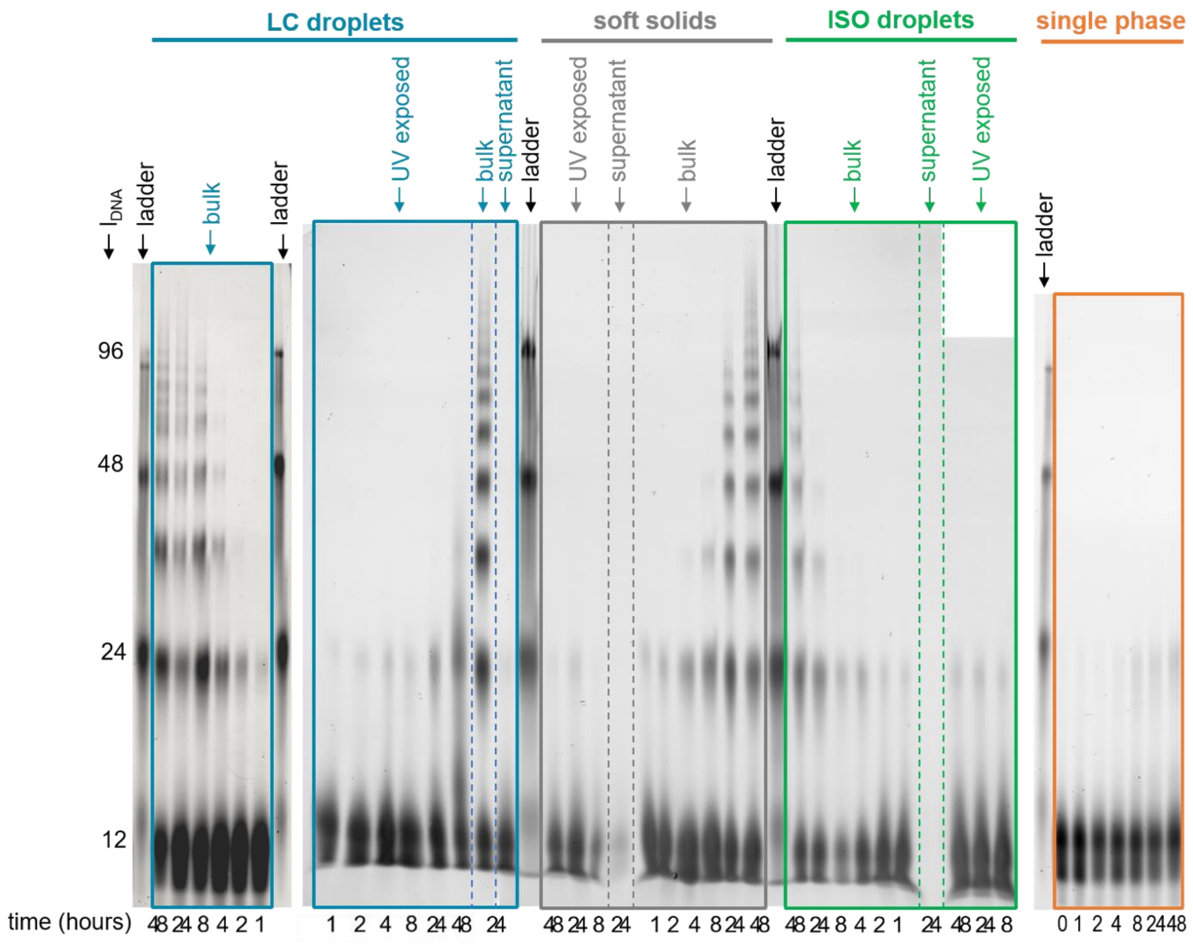

Supplementary Figure 13. Time-dependent full polyacrylamide electrophoresis gels. Full gel images of a 15% PAGE run of the ligation product obtained in DDp/*trans*-azoTAB solutions (10 mM total charge concentration) for different reaction conditions and in different phases, as indicated. Reaction times, from 0 to 48 hours, are reported below the corresponding lanes. Grey, cyan, green and orange boxes refer to reactions carried out on soft solids (0 mM NaCl + 80 mM EDC), LC droplets (100 mM NaCl + 80 mM EDC), ISO droplets (200 mM NaCl + 80 mM EDC), and single phase (400 mM NaCl + 80 mM EDC), respectively. “Bulk” refers to analyses of the dense phase after centrifugation and supernatant removal; “supernatant” refers to analyses of the associated supernatant phase; “UV exposed” refers to samples continuously exposed to UV light during ligation (please note these lanes under UV light were not used in the main text but are just shown here to avoid cropping the full gel images). Analyses of the supernatant show no significant extent of reaction for up to 24h reaction time. Please note that the gel lane for the LC supernatant shows a higher concentration of unreacted DD compared to supernatants from soft solids of ISO coacervates. This is due to a 10-fold higher loading of the gels to ensure no bands are detected at high polymerisation degrees. Ladders are constituted by mixing of 12-, 24-, 48- and 96-mer DNA oligomers synthesized with repetition of the 12-mer DD sequence.

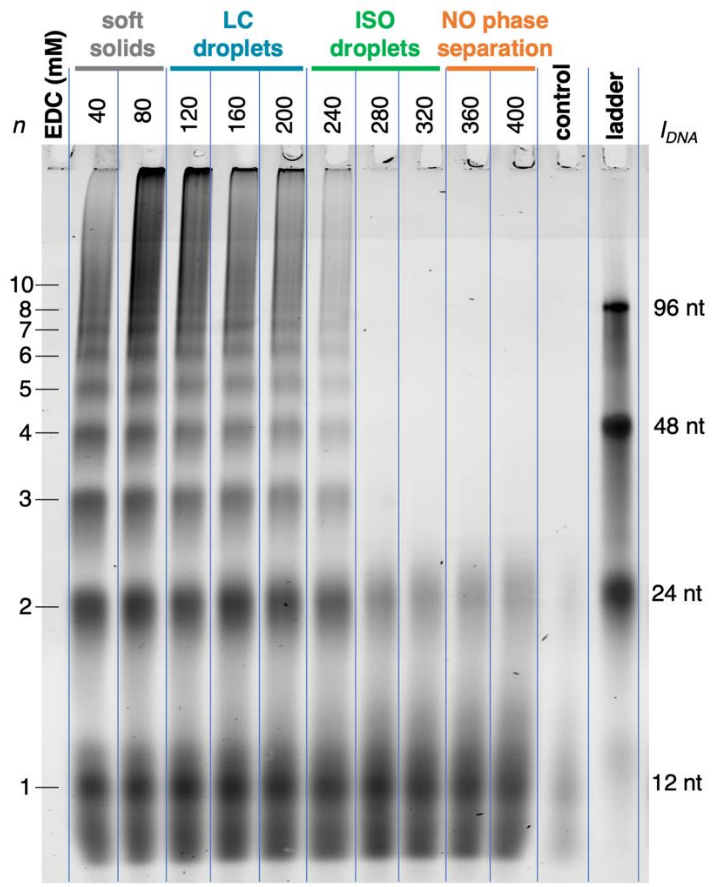

Supplementary Figure 14. Influence of EDC concentrations on ligation. Full gel image of a 15% PAGE run of the ligation product obtained in DDp/*trans*-azoTAB solutions (10 mM total charge concentration) at increasing EDC concentration between 40 and 400 mM (values above each lane). The phases formed in each condition (aggregates, soft solids, LC or ISO coacervates droplets, or no phase separation), as observed by optical microscopy imaging at the beginning of the reaction, are indicated at the top for reference. All reactions lasted 24 hours. Long DNA polymers are prevalently produced at lower values of EDC concentration, corresponding to the presence of soft solids and/or LC coacervates, while decrease of long polymers production is observed at increasing total EDC concentrations, corresponding to ISO coacervates and single-phase conditions. An abrupt decrease in ligation efficiency is observed in proximity of the critical ionic strength for DDp/*trans*-azoTAB coacervation, conditions at which the volume fraction of the dense coacervate phase dramatically decreases.

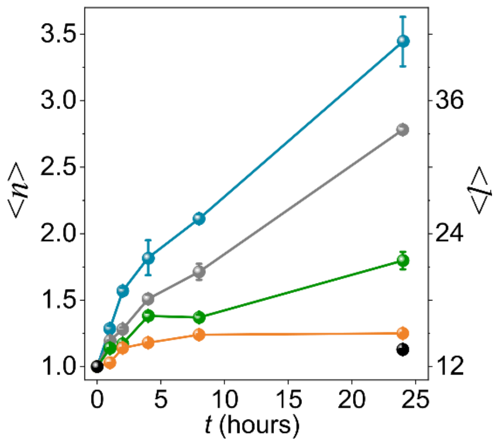

Supplementary Figure 15. Average degree of polymerization during oligonucleotide ligation. Plots showing the change of the average degree of polymerization, $<n>$, and average chain length expressed in number of nucleotides, $<l>$, at different times during the ligation reaction in DDp/*trans*-azoTAB solutions (10 mM total charge concentration) in different phases (grey: soft solids (0 mM NaCl + 80 mM EDC); cyan: LC droplets (100 mM NaCl + 80 mM EDC); green: ISO droplets (200 mM NaCl + 80 mM EDC); orange: single phase (400 mM NaCl + 80 mM EDC); black: supernatant obtained from centrifuged LC droplets). Solid lines are guides to the eye. Values were extracted by analysing samples from the same experiment (*n*=1) run in the same PAGE gel (except for single phase, whose gel was run in parallel) shown in Supplementary Fig. 13 using two fitting procedures, as detailed in Supplementary Note 3, and are reported as mean ± SD of the two values obtained from fits. Error bars thus represent errors associated to uncertainties in the fits.

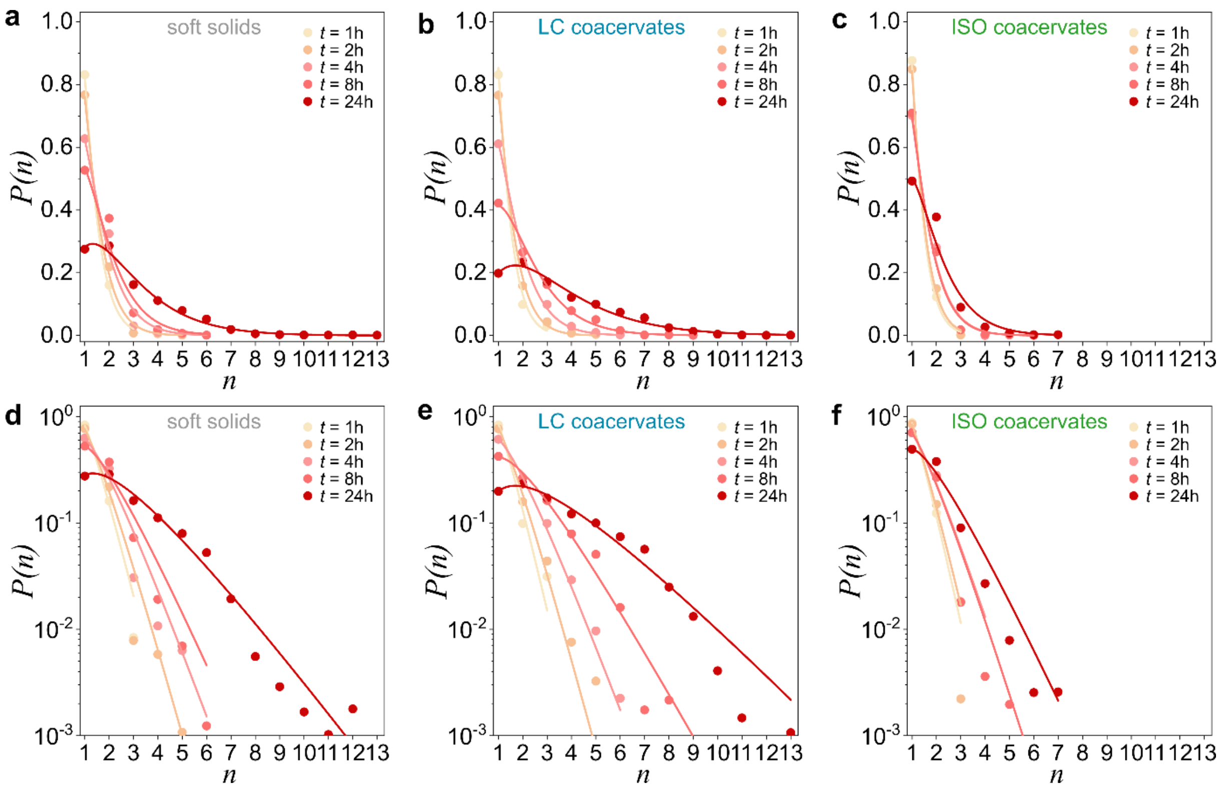

Supplementary Figure 16. Product distribution during oligonucleotide ligation. a-**f**, Plots showing the change of the weight fraction product distributions, $P(n)$, at different times during the ligation reaction in DDp/*trans*-azoTAB solutions (10 mM total charge concentration) prepared as soft solids (0 mM NaCl + 80 mM EDC, **a**,**d**), LC coacervate droplets (100 mM NaCl + 80 mM EDC, **b**,**e**) or ISO coacervate droplets (200 mM NaCl + 80 mM EDC, **c**,**f**). Note that **d**-**f** show the same results as **a**-**c** but with a logarithmic *y*-scale rather instead of a linear scale to better show the weight fractions of each polymer product. Data points were obtained by analysing the gels shown in Supplementary Fig. 13 (see Supplementary Note 3). Solid lines are fit of the data points with a Flory's model for linear polymer condensation (Supplementary Note 2).

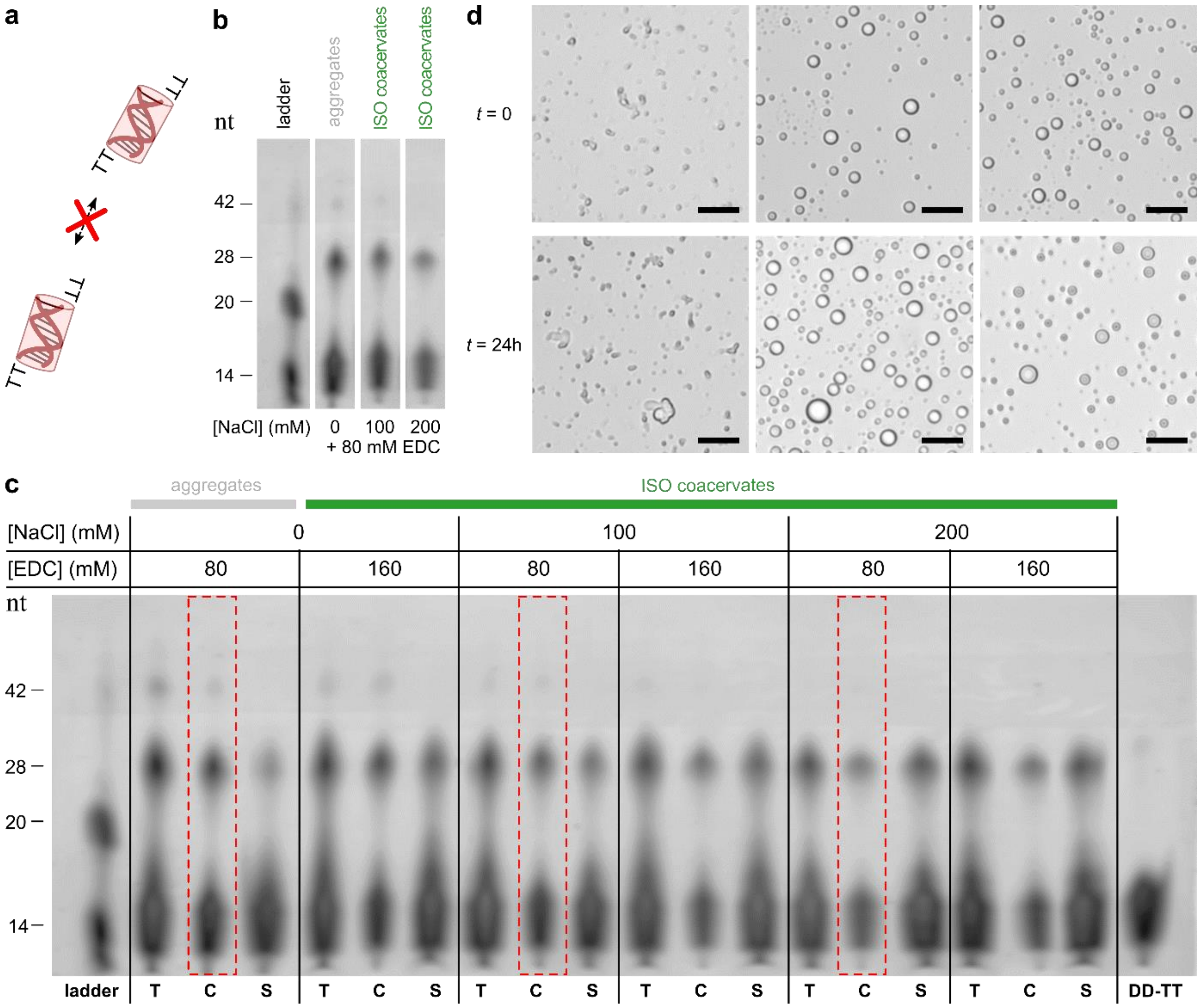

Supplementary Figure 17. Ligation experiments with DD-TTp duplexes. a, Schematic representation of DD-TT duplexes, where the 3'-TT dangling-ends inhibit end-to-end stacking interactions. **b**, **c**, Cropped (**b**) and full (**c**) gel images of a 15% PAGE run of the ligation product obtained after 24 hours DD-TTp/*trans*-azoTAB solutions (equimolar charge mixtures, 10 mM total charge concentration) in different phases, as labelled. The concentrations of NaCl and EDC used for each condition are specified. In the full gel image, "T", "C" and "S" stand for total solution, dense coacervate phase and supernatant, respectively; "DD-TT" shows the electrophoretic mobility of unreacted DD-TTp oligonucleotide. Ladder is composed by unreacted 12-TTp and a 20 bp oligonucleotide. Red dotted regions show the gel lanes that were used in **b**. **d**, Bright-field optical microscopy images of charged-balanced reactive mixtures of DD-TTp (5 mM nucleobase concentration) and *trans*-azoTAB (5 mM) prepared at different total ionic strength (aggregates: 80 mM EDC; ISO droplets: 100 or 200 mM NaCl + 80 mM EDC) at $t = 0$ and after 24 hours, as labelled, showing no morphological change. Scale bars, 10 µm.

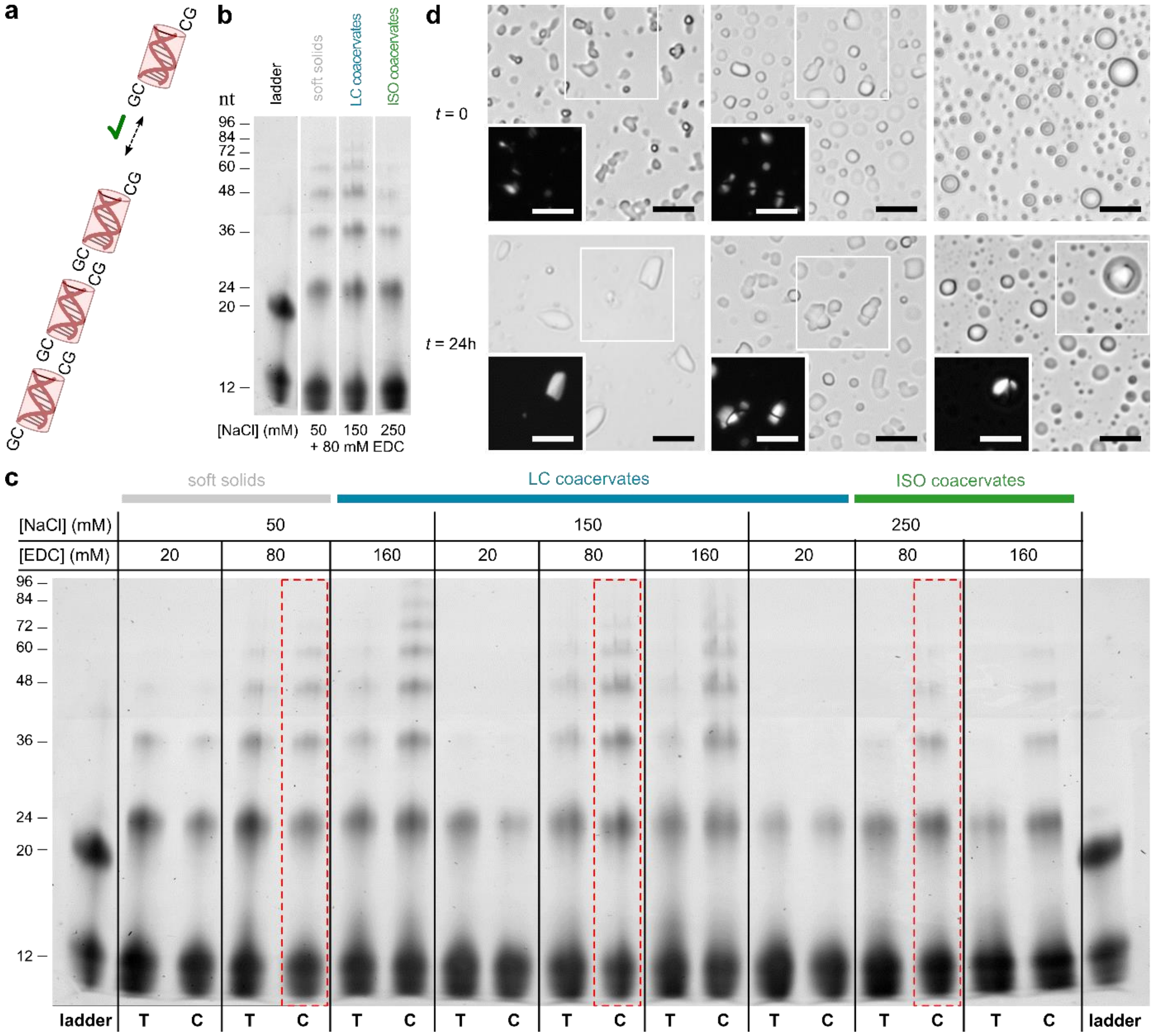

Supplementary Figure 18. Ligation experiments with 12-CGp duplexes. a, Schematic representation of 12-CG duplexes, where the 3'-CG sticky ends favour end-to-end pairing and stacking interactions. **b**, **c**, Cropped (**b**) and full (**c**) gel images of a 15% PAGE run of the ligation product obtained after 24 hours 12-CGp/*trans*-azoTAB solutions (equimolar charge mixtures, 10 mM total charge concentration) in different phases, as labelled. The concentrations of NaCl and EDC used for each condition are specified. In the full gel image, "T" and "C" stand fortotal solution and dense coacervate phase, respectively. Ladder is composed by unreacted 12-GCp and a 20 bp oligonucleotide. Red dotted regions show the gel lanes that were used in **b**. **d**, Bright-field optical microscopy images of charged-balanced reactive mixtures of 12-CGp (5 mM nucleobase concentration) and *trans*-azoTAB (5 mM) prepared at different total ionic strength (soft solids: 50 mM NaCl + 80 mM EDC; LC droplets: 150 mM NaCl + 80 mM EDC; ISO droplets: 250 mM NaCl + 80 mM EDC) at $t = 0$ and after 24 hours, as labelled. Insets show the white squared areas under 90° crossed polarisers. Scale bars, 10 µm. Images show transitions similar as for DDp, such as the formation of LC-in-ISO multiphase coacervates starting from ISO droplets.

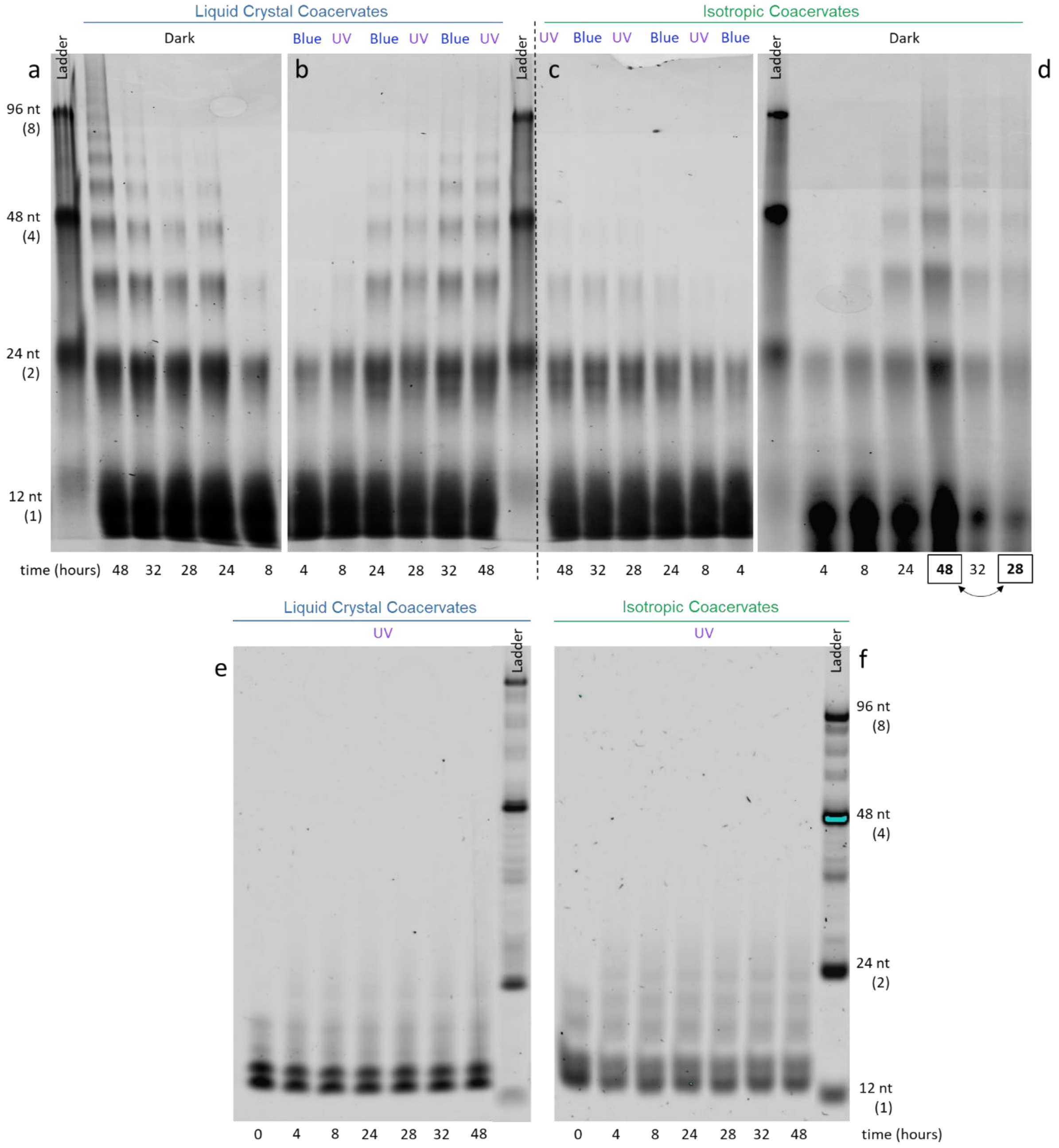

Supplementary Figure 19. Polyacrylamide gel electrophoresis for dark and UV/blue cycles. a-d, Full gel images of the DDp ligation products obtained from reactions performed in the dark or under UV/blue light cycles for LC and ISO coacervates. Reactions were stopped after 4, 8, 24, 28, 32 and 48 hours, as indicated below the gels, which corresponds to the timings of blue/UV switch. Cyan, green and purple rectangles and numbers help to identify the highest polymerization degree products observable in a gel lane. Conditions are as follows: **a**, DDp ligation in LC coacervates (100 mM NaCl + 80 mM EDC) kept in dark for the whole experiment duration. **b**, DDp ligation in LC coacervates exposed to UV/blue light cycles. **c**, DDp ligation in ISO coacervates (200 mM NaCl + 80 mM EDC) kept in dark for the whole experiment duration. **d**, DDp ligation in ISO coacervates exposed to light UV/blue cycles. **e,f,** DDp ligation in LC (**e**) or ISO (**f**) coacervates constantly exposed to UV light. Note that PAGE in **e**,**f** show doubling of the 12nt band and intermediate bands between 12nt and 24nt which don't correspond to reaction products but are artefacts of the gel run, since the same pattern is shown by the t = 0h control lane. These observations do not impede the analysis of the gels to extract the reaction yield.

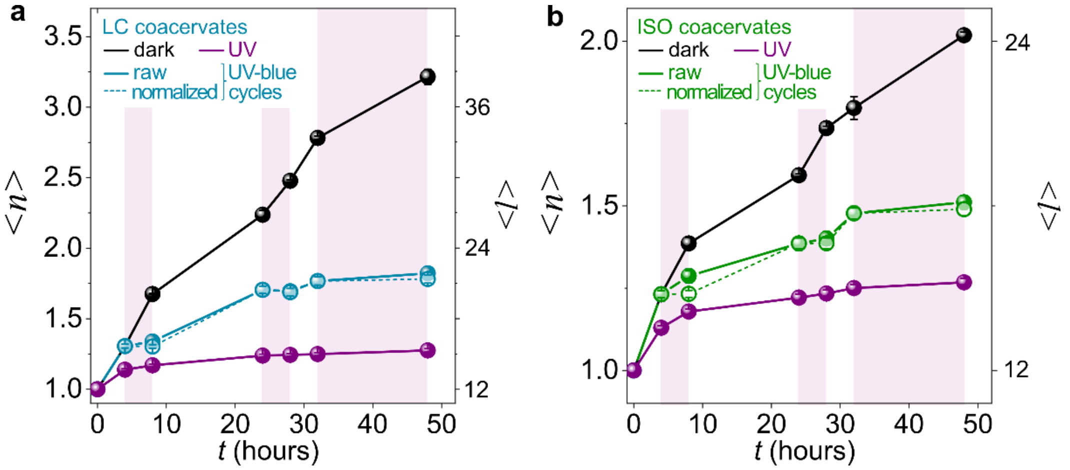

Supplementary Figure 20. Results of DDp ligation as a function of time for reactions performed in the dark, under UV or using UV/blue cycles. a,**b**, Average degree of polymerization, <*n*>, and corresponding average number of nucleobases in polynucleotides, <*l*>, calculated from fitted products distributions (see Supplementary Notes 2 and 3) for reactive LC (**a**) and ISO (**b**) coacervate droplets kept in the dark (black dots) or under constant UV light (purple dots), or exposed to cycles of UV and blue light (cyan or green dots). Lines are guides to the eye. Data was obtained from analysis of PAGE runs shown in Supplementary Fig. 19. Normalized data for UV/blue cycles (open dots, dotted line) was obtained by subtracting the contribution of ligation occurring during UV exposure measured in dilute samples constantly exposed to UV light. Solid lines are guides to the eye. Values were extracted by analysing samples from the same experiment (n=1) run in the same PAGE gel shown in Supplementary Fig. 19 using two fitting procedures, as detailed in Supplementary Note 3, and are reported as mean ± SD of the two values obtained from fits. Error bars thus represent errors associated to uncertainties in the fits.

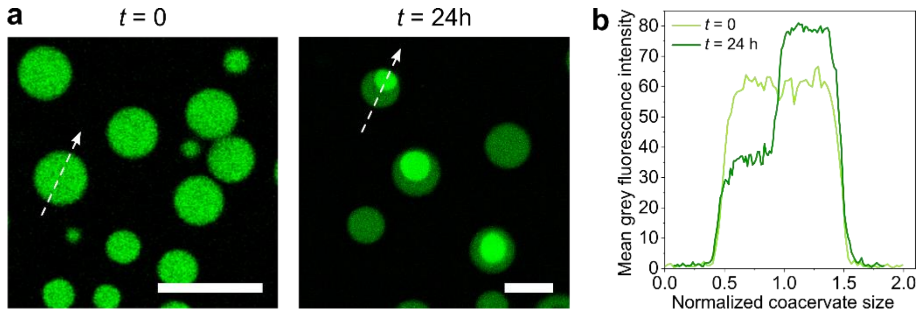

Supplementary Figure 21. Oligonucleotide elongation produces multiphase coacervates. a, Confocal fluorescence microscopy images of DDp/trans-azoTAB ISO coacervate droplets produced at 10 mM total charge concentration in the presence of 200 mM NaCl and 80 mM EDC at t = 0 and after 24 hours, showing the formation of a bright sub-domain over time. Scale bars, 10 µm. **b**, Fluorescent line profile across a single coacervate droplet at t = 0 and t = 24 h (white dashed arrows in **a**).

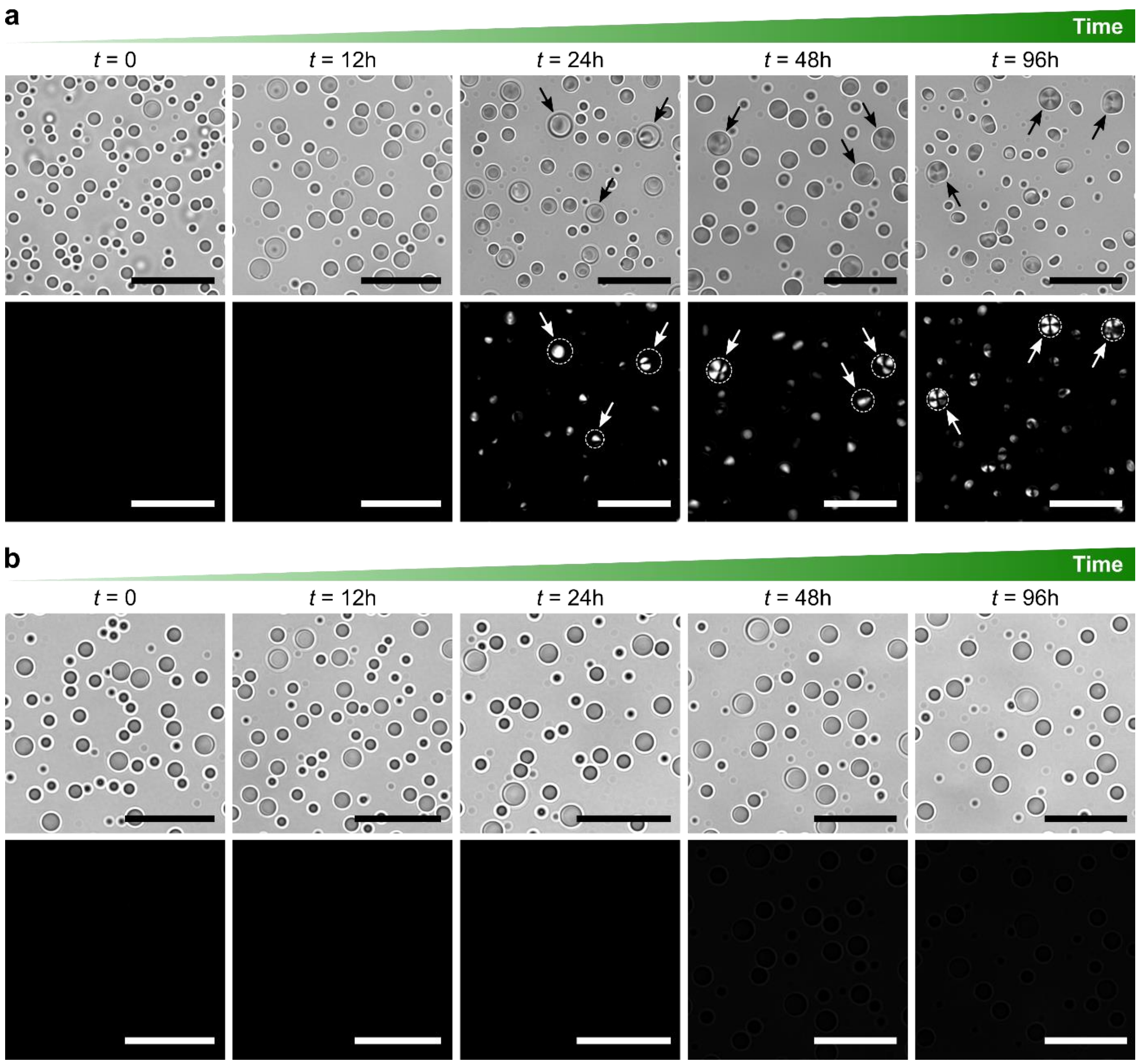

Supplementary Figure 22. Imaging of ISO coacervates over reaction time. a,b, Time-dependent bright-field (top row) and 90°-polarized (bottom row) optical microscopy images of reactive DDp/*trans*-azoTAB coacervates (**a**) or unreactive DD/*trans*-azoTAB coacervates (**b**) prepared at equimolar charge ratio (5 mM) in the presence of 200 mM NaCl and 80 mM EDC. In **a**, a gradual formation of birefringent sub-phases is observed in the initial fully isotropic droplets reactive coacervates, associated to a transition to LC columnar phases. After 96 hours, the whole droplets exhibit a LC columnar phase and start changing morphology to less spherical droplets. Arrows point to examples of droplets containing birefringent sub-domains (at *t* = 24h and 48h) or appearing fully birefrigent (at *t* = 96 h). In comparison, control experiments in **b** show no morphological or structural changes of unreactive ISO coacervates over time. Scale bars, 20 µm.

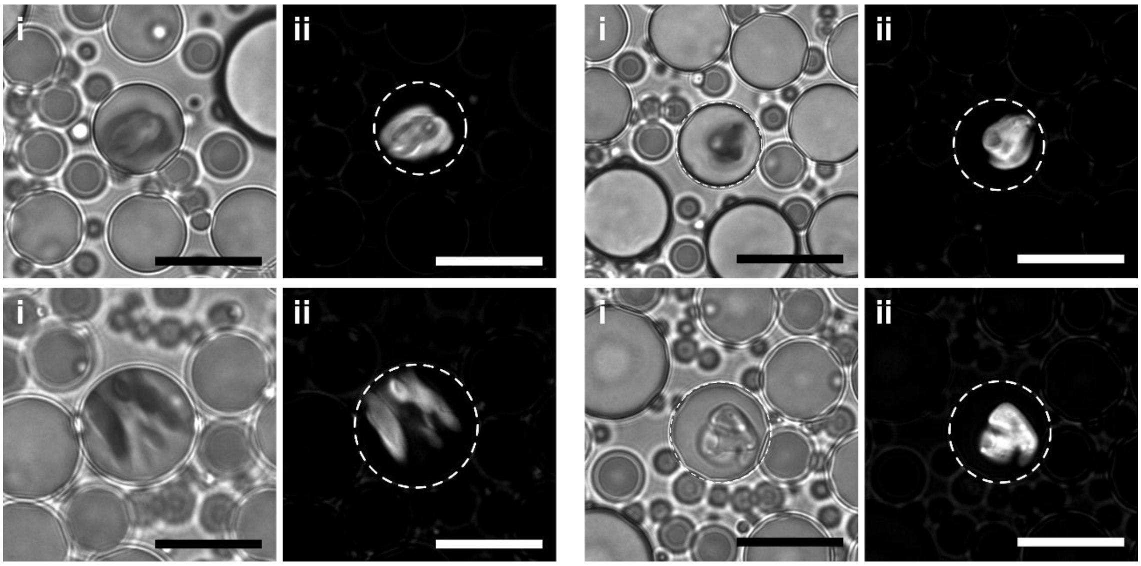

Supplementary Figure 23. Mixed oligonucleotide lengths form multiphase LC-in-ISO coacervate droplets with *trans*-azoTAB. Bright-field (i) and corresponding 90°-polarized (ii) optical microscopy images of 70 mol% DD monomer and 30 mol% DD_2 dimer (30 mM total nucleobase concentration) mixed with *trans*-azoTAB at equimolar charge ratio in the presence of 350 mM NaCl. Scale bars, 20 µm. Under this ionic strength condition, DD/*trans*-azoTAB mixtures form ISO coacervate droplets. Here, we observe that a few droplets contain a birefringent sub-phase, which we attributed to complexes between the longer DD_2 strands and *trans*-azoTAB. Note that these experiments were performed at 30 mM total nucleobase concentration to favour the formation of large droplets and better observe the internal sub-phase.

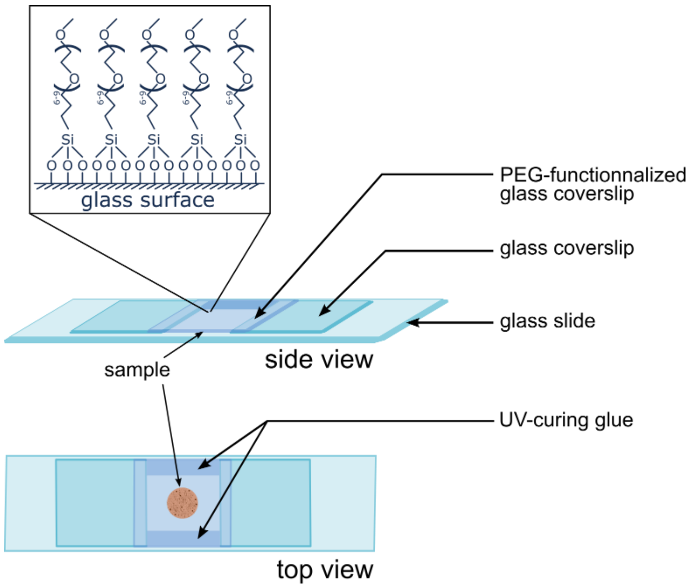

Supplementary Figure 24. **Schematic view of the custom-made capillary chamber used to image coacervate droplets.** Glass coverslips were first glued onto a glass slide using UV-curing glue. PEGylation was performed as detailed in the methods. The PEGylated coverslip was glued on top of the other two coverslips using UV-curing glue, and sealed at both ends after sample loading. Note that imaging was performed on an inverted microscope through the PEGylated coverslip.

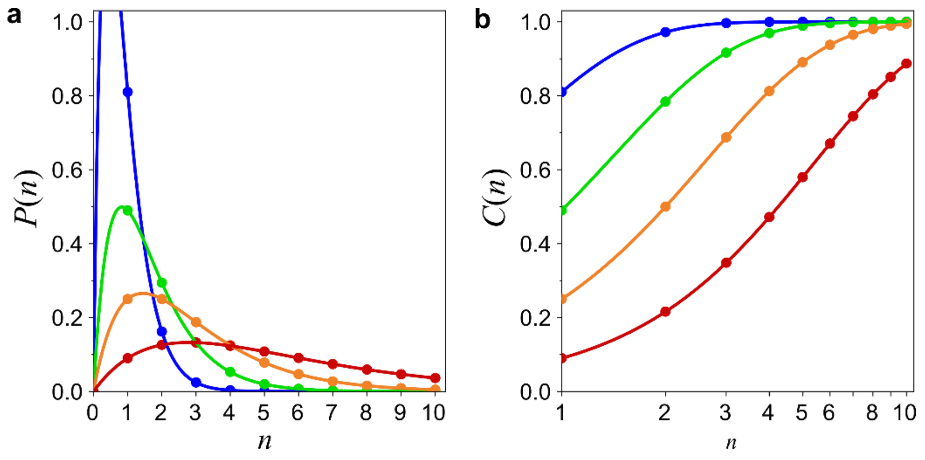

Supplementary Figure 25. **Plots of the linear condensation polymers distributions from Flory's theory.** **a**, Weight fraction distribution, $P(n)$ from Supplementary Eq. 2, for four different polymerization yields, p = 0.1, 0.3, 0.5, 0.7, showing that the maximum of the products distribution moves towards higher n as the yield grows, while the fraction of monomers, $P(1)$, decreases. **b**, Cumulative weight fraction distribution, $C(n)$, from Supplementary Eq. 3, for the same yields. $C(n) = i$, where $0 < i < 1$, gives the maximum length of the polymers that are in the *ix100-th* percentile.

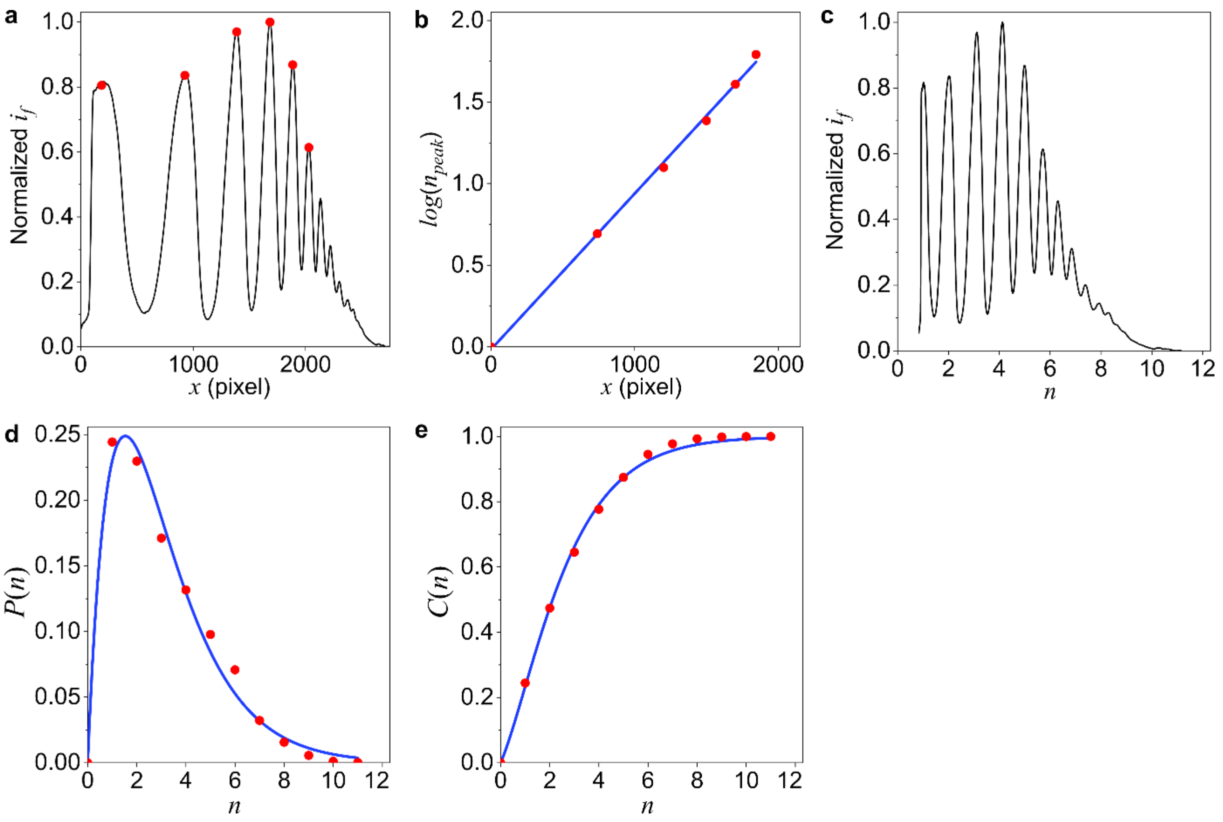

Supplementary Figure 26. **Illustrative steps of the analysis of electrophoretic polyacrylamide gels**. We show here an example of analysis for the electrophoretic run obtained from a 24 hours ligation reaction in LC coacervates (shown in Fig. 2a). **a**, Fluorescence intensity i_f vs. position x in pixel after background subtraction and normalization. Well-defined peaks are automatically identified by the Matlab program (red dots). **b**, Logarithm of the peaks order, $log(n_{peaks})$, as a function of the peak position (red dots). The blue line indicates the best linear fit, which allows the conversion between x and degree of polymerization n. **c**, Fluorescence intensity rescaled as a function of the degree of polymerization. **d**, Products weight distribution $P(n)$ (red dots) obtained from the normalized integral of each peak, fitted by the Flory model for simple polymerization (blue line). **e**, Cumulative weight distribution $C(n)$ (red dots) fitted by the Flory model (blue line).

Supplementary Notes

Supplementary Note 1. Estimation of the oligonucleotide concentration in dark-adapted ISO coacervate droplets.

The concentration of DD strands in dark-adapted ISO coacervate droplets was estimated using the relative fraction of DD in the dense coacervate phase vs. supernatant determined by UV-vis spectroscopy (see methods). The missing information to extract absolute concentrations is the relative volume fraction of dense coacervate phase vs. supernatant. Due to the low amount of sample produced, we could not rely on bulk volume measurements, e.g. via pipetting. Rather, we estimated these volume fractions using microscopy imaging, as detailed below.

First, 1 µL of *trans*-azoTAB/DD ISO coacervates prepared at 350 mM NaCl was pipetted in a custom-made capillary chamber (Supplementary Fig. 24) that was sealed with UV-curing glue (by protecting the sample from UV light irradiation using foil). Coacervate droplets were left to settle for 30 minutes on the PEGylated coverslip. Images of the whole sample were taken on an inverted Leica DMI 4000B microscope using a ×5 air objective, and the area of the sample was measured using ImageJ to estimate the thickness of our observation chamber (knowing the total volume added to the chamber). Typical values were in the range of 120-150 µm.

Images of coacervate droplets were then acquired using a ×63 oil immersion objective, and the total volume of coacervate phase in the field of view estimated by measuring the radius of all the coacervate droplets, assuming they adopted a perfect spherical shape. The total volume of the field of view was also determined using the above-estimated thickness of the observation chamber, and used to infer the volume fraction of ISO coacervate phase vs. supernatant. This volume fraction was finally used to estimate the concentration of DD in the bulk coacervate phase vs. supernatant solution, using the fraction of DD measured in each phase. Such measurements were repeated on 5 different samples and the average value and standard deviation reported.

Note that such an estimation could not be performed for LC coacervate droplets or soft solids due to the non-sphericity of most of the complexes. However, the typical phase diagram of associative phase separation suggests that the oligonucleotide concentration in phases produced at lower ionic strength (here soft solids and LC coacervates) would be higher than at higher ionic strength.

Supplementary Note 2. Flory's theory for linear condensation of polymers.

Flory's theory offers a simple theoretical tool to describe the molecular size distribution of polymers formed by condensation of bifunctional compounds.[4] Here, we used the results of the Flory model to fit the experimental products distributions obtained from ligation reactions.

Given a polymerization process starting from an initial number of monomeric reactants, N_0, and given N the number of unreacted monomers at a certain reaction extent, the probability to produce polymers composed of n monomers (n-mer) is:

$$\pi(n) = p^{n-1}(1-p)^2 \qquad (\textit{Equation 1})$$

where p is the fraction the total number of reacted monomers $p = (N_0 - N) / N_0$, which thus corresponds to the reaction yield.

Since each n-mer is composed of n monomers, the fraction of monomers that are part of n-mers over the total number of monomers, i.e. the weight fraction distribution is:

$$P(n) = n\, p^{n-1}(1-p)^2 \qquad (\textit{Equation 2})$$

from which it is straightforward to calculate the cumulative weight fraction distribution:

$$C(n) = \sum_{i=0}^{n} P(i) = 1-(1+n)p^n + np^{n+1} \qquad (\textit{Equation 3})$$

From the weight fraction distribution, it is possible to obtain the average degree of polymerization, $<n>$, as:

$$\langle n \rangle = \frac{\sum nP(n)}{\sum P(n)} = \frac{\sum n^2\, p^{n-1}(1-p)^2}{\sum n\, p^{n-1}(1-p)^2} = \frac{1+p}{1-p} \qquad (\textit{Equation 4})$$

Representative examples of plots of $P(n)$ and $C(n)$ are given on Supplementary Fig. 25.

Supplementary Note 3. Analysis of the fluorescence intensity profiles of PAGE runs.

Gray scale 16-bit images of the gels were processed with MacBiophotonics ImageJ software. Brightness adjustments were made to maximize the signal-to-background ratio, avoiding saturation of the image. The intensity profiles of each lane were extracted by averaging on rectangular areas covering the full width of the gel lane. Analyses of the intensity profiles were performed using a Matlab program as follows:

- Fluorescence intensity profiles $i_f(x)$, where x is the pixel position on the image, were corrected by background subtraction (measured as the fluorescence intensity before the first peak) and normalized with respect to the highest peak (Supplementary Fig. 26a).
- By assuming a linear dependence of the electrophoretic mobility on the logarithm of the strand length to be valid in the whole gel, we could rescale the x-axis into an axis indicating the degree of polymerization, n. Accordingly, for each profile, the program identified the positions x_{peaks} of well-defined intensity peaks (red dots in Supplementary Fig. 26a). The logarithm $log(n_{peak})$, where n_{peak} is the peak order, was plotted against x_{peaks} and fitted with a linear function (Supplementary Fig. 26b) that was used to convert the x-axis into the scale of the degree of polymerization $i_f(n)$ (Supplementary Fig. 26c), where the variable n is considered continuous.
- By virtue of the interaction of SYBR Gold with *ds*DNA strands, the intensity in the gel bands is proportional to the total mass of nucleic acids they contain and could thus be compared with the weight fraction distributions introduced in Supplementary Note 2. To this aim, we first calculated the integral of the fluorescence intensity:

$$I(n) = \frac{1}{I_0}\int_0^n i_f(x)dx$$

(*Equation 5*)

where $I_0 = \int_0^\infty i_f(x)dx$. $I(n)$ represents the mass of the ligation products up to n. To obtain discretized quantities that can be compared to $P(n)$ and $C(n)$ derived from Flory's theory (as discussed in Supplementary Note 2), we segmented the integration and calculated:

$$P(n) = \frac{1}{I_0}\int_{n-0.5}^{n+0.5} i_f(x)dx$$

(*Equation 6*)

which we plotted in Supplementary Fig. 26d (red dots). We also calculated:

$$C(n) = \frac{1}{I_0}\int_0^{n+0.5} i_f(x)dx = \sum_{n'=0}^{n} P(n')$$

(*Equation 7*)

which we plotted in Supplementary Fig. 26e (red dots).

- The experimental $P(n)$ and $C(n)$ were then fitted using the corresponding functions from the Flory model (blue lines in Supplementary Figs. 26d,e, from Supplementary Equations 2 and 3, respectively) to obtain the reaction yield p. Two values of p were obtained independently from fitting $P(n)$ and $C(n)$, and the average value and associated error (reflecting the uncertainty of the fits) reported.
- Average degrees of polymerization, $\langle n \rangle$, could last be extracted using Equation 4.

Supplementary Note 4. Ligation kinetics.

The condensation reaction of 3’-phosphate DNA oligomers via EDC activation involves the formation of a reactive intermediate (with a rate k_1) followed either by a nucleophilic substitution with a DNA 5’-hydroxyl terminus (rate k_L) or hydrolysis of the activated intermediate (rate k_2).[5,6] Direct hydrolysis of unreacted EDC to urea (rate k_h) has to be taken into account too.

Previous studies have independently measured the values of k_1 = 0.020 h^{-1} mM^{-1} and k_h = 0.033 h^{-1} (ref. 7). The full ligation reaction scheme is defined by the corresponding set of differential equations which can be numerically solved to evaluate the best k_2/k_L ratio to fit the experimental data. Such an approach requires further (and more or less arbitrary) assumptions when considering a phase separated system as in the case of coacervates.[6] For the sake of simplicity and with our principal aim to compare the reaction yields in different coacervate phases, we decided in our analysis to treat the reaction as a catalysed step-growth polyesterification and extracted only a coarse estimation of the overall reaction rate. This approximation can be justified considering that:

- EDC hydrolysis is very slow (with a characteristic time of 30 hours if we take the reported k_h = 0.033 h^{-1}) and EDC is added in large excess compared to the terminal phosphate groups ([EDC]/[DDp] = 160), so the concentration of EDC can be considered as almost constant;
- the concentration of DDp in coacervates is very high, so the activation step is very fast (if we take the published k_1 (k_1 = 0.020 h^{-1} mM^{-1}) and our measured concentration of DDp in ISO coacervates ([DDp] = 130 mM), the characteristic activation time would be 0.38 h = 23 minutes);
- water activity in the dense coacervate phase is lower than in the supernatant, so hydrolysis of the activated phosphate is presumably slower, and DDp strands are in close proximity, so we can assume that the formation of a phosphodiester bond (k_L) is favoured over hydrolysis (k_2). This is supported by literature data for which k_2/k_L ~ 10^{-2} (ref. 6).

Based on these assumptions, we could use the simple equation of catalysed step-growth polymerization, which is a second-order law that gives as a general expression:

$$p(t) = A\left(1 - \frac{1}{1+t/\tau}\right) \qquad (\textit{Equation 8})$$

where $\tau = 1/(k[P]_0)$, where $[P]_0$ is the initial molar concentration of terminal phosphate (which is here is equal to the concentration of DD oligomers).

We used this expression to fit the experimental data shown on Fig. 2c, using a fixed maximum achievable reaction yield, A, as the value that better fitted the data for ligation in LC coacervates (A = 0.63), which is the experimental condition showing the highest yield. The fits provided characteristic times τ_{LC} = 4.5h for liquid crystals, $\tau_{soft\ solids}$ = 9h for soft solids and τ_{ISO} = 22h for isotropic coacervates. Thus, the reaction was 2×~~,~~ and 5× faster in LC coacervates~~,~~ compared to soft solids and ISO coacervates, respectively.

Supplementary references

[1] Lafon, S., Martin, N. Reversible photocontrol of DNA coacervation. *Methods Enzymol.*, **646***,* 329-351 (2020)

[2] Fraccia, T.P., Zia, T.J. Liquid crystal coacervates composed of short double stranded DNA and cationic peptides. *ACS Nano*, **14**, 15071-15082 (2020)

[3] Düster, R., Kaltheuner, I.H., Schmitz, M., Geyer, M., 1,6-Hexanediol, commonly used to dissolve liquid–liquid phase separated condensates, directly impairs kinase and phosphatase activities. *J. Biol. Chem.*, **296**, 100260 (2021)

[4] Flory, J. Molecular size distribution in linear condensation polymers. *J. Am. Chem. Soc.* **1,** 1877–1885 (1936)

[5] Grabarek, Z., Gergely, J., Zero-length crosslinking procedure with the use of active esters. *Anal. Biochem.*, **185**, 131-135 (1990)

[6] Obianyor, C., Newmann, G., Clifton, B.E., Grover, M.A., Hud, N.V., Towards efficient nonenzymatic DNA ligation: comparing key parameters for maximizing ligation rates and yields with carbodiimide activation. *ChemBioChem*, **21**, 3359-3370 (2020)

[7] Todisco, M., Fraccia, T.P. *et al.*, Nonenzymatic polymerization into long linear RNA templated by liquid crystal self-assembly. *ACS Nano*, **12**, 9750-9762 (2018)
