# Supplementary material for: Non-enzymatic oligonucleotide ligation in coacervate protocells sustains compartment-content coupling
Source: Nat Commun. 2023 May 9;14:2606. doi: 10.1038/s41467-023-38163-8 (PMC10169843; doi:10.1038/s41467-023-38163-8)
Supplement: Supplementary file 2 — Description of Additional Supplementary Files [file 41467_2023_38163_MOESM2_ESM.pdf]

Description of Additional Supplementary Files

Supplementary Movie 1. Optical microscopy video showing dissolution of *trans*-azoTAB/DD coacervate micro-droplets under UV light. Movie is shown at real-time speed at 10 frames per second.

Supplementary Movie 2. Optical microscopy video showing coacervate formation in a UV-equilibrated *cis*-azoTAB/DD solution under blue light. Movie is shown at ×2 (from t = 0 to t = 7s) then ×8 (from t = 7s onwards) real-time speed at 8 frames per second.

Supplementary Movie 3. Optical microscopy video showing UV light-mediated dissolution of the outer ISO coacervate phase in *trans*-azoTAB/DDp LC-in-ISO multiphase coacervate micro-droplets obtained after 24 h of reaction with EDC during UV/blue light cycles. Movie is shown at real-time speed at 5 frames per second.

Supplementary Movie 4. Optical microscopy video showing blue light-mediated reformation of an ISO coacervate phase around LC domains produced after 28 h of reaction with EDC just after UV exposure during UV/blue light cycles. Movie is shown at ×5 real-time speed at 50 frames per second.
