# Supplementary material for: Non-enzymatic oligonucleotide ligation in coacervate protocells sustains compartment-content coupling
Source: Nat Commun. 2023 May 9;14:2606. doi: 10.1038/s41467-023-38163-8 (PMC10169843; doi:10.1038/s41467-023-38163-8)
Supplement: Supplementary file 7 — Reporting Summary [file 41467_2023_38163_MOESM7_ESM.pdf]

nature portfolio

Corresponding author(s): Nicolas MARTIN, Tommaso FRACCIA

Last updated by author(s): Apr 6, 2023

Reporting Summary

Nature Portfolio wishes to improve the reproducibility of the work that we publish. This form provides structure for consistency and transparency in reporting. For further information on Nature Portfolio policies, see our Editorial Policies and the Editorial Policy Checklist.

Statistics

For all statistical analyses, confirm that the following items are present in the figure legend, table legend, main text, or Methods section.

n/a	Confirmed	
☐	☒	The exact sample size (*n*) for each experimental group/condition, given as a discrete number and unit of measurement
☐	☒	A statement on whether measurements were taken from distinct samples or whether the same sample was measured repeatedly
☒	☐	The statistical test(s) used AND whether they are one- or two-sided<br>*Only common tests should be described solely by name; describe more complex techniques in the Methods section.*
☒	☐	A description of all covariates tested
☒	☐	A description of any assumptions or corrections, such as tests of normality and adjustment for multiple comparisons
☐	☒	A full description of the statistical parameters including central tendency (e.g. means) or other basic estimates (e.g. regression coefficient) AND variation (e.g. standard deviation) or associated estimates of uncertainty (e.g. confidence intervals)
☒	☐	For null hypothesis testing, the test statistic (e.g. *F*, *t*, *r*) with confidence intervals, effect sizes, degrees of freedom and *P* value noted<br>*Give P values as exact values whenever suitable.*
☒	☐	For Bayesian analysis, information on the choice of priors and Markov chain Monte Carlo settings
☒	☐	For hierarchical and complex designs, identification of the appropriate level for tests and full reporting of outcomes
☒	☐	Estimates of effect sizes (e.g. Cohen's *d*, Pearson's *r*), indicating how they were calculated

Our web collection on statistics for biologists contains articles on many of the points above.

Software and code

Policy information about availability of computer code

Data collection	MicroManager (v1.4); Nikon NIS-Elements AR (v5.30.03); Leica Application Suite LAS X (v3.5.7.23225); SoftMax Pro (v6.2.2)
Data analysis	Origin 2019, 9.6.0.172, Academic (OriginLab)<br>FIJI (ImageJ) v1.53f (https://imagej.net/Fiji)<br>MathWorks Matlab R2022a (https://fr.mathworks.com/products/matlab.html)

For manuscripts utilizing custom algorithms or software that are central to the research but not yet described in published literature, software must be made available to editors and reviewers. We strongly encourage code deposition in a community repository (e.g. GitHub). See the Nature Portfolio guidelines for submitting code & software for further information.

Data

Policy information about availability of data

All manuscripts must include a data availability statement. This statement should provide the following information, where applicable:

- Accession codes, unique identifiers, or web links for publicly available datasets
- A description of any restrictions on data availability
- For clinical datasets or third party data, please ensure that the statement adheres to our policy

The data supporting the findings of this study are available within the article and its Supplementary Information. Numeric data underlying all plots and gels in the main publication are available as Source Data files. All other data is available from the corresponding author upon request. Source data are provided with this paper.

Human research participants

Policy information about studies involving human research participants and Sex and Gender in Research.

Reporting on sex and gender	n/a
Population characteristics	n/a
Recruitment	n/a
Ethics oversight	n/a

Note that full information on the approval of the study protocol must also be provided in the manuscript.

Field-specific reporting

Please select the one below that is the best fit for your research. If you are not sure, read the appropriate sections before making your selection.

☒ Life sciences ☐ Behavioural & social sciences ☐ Ecological, evolutionary & environmental sciences

For a reference copy of the document with all sections, see nature.com/documents/nr-reporting-summary-flat.pdf

Life sciences study design

All studies must disclose on these points even when the disclosure is negative.

Sample size	No statistical method was used to determine the sample size. In order to avoid quantitative comparisons between samples on different gels, reaction yields were derived from analysis of lanes belonging to the same gel (n=1), for which we used two different fitting methods. Values are reported as mean ± SD of the 2 values obtained from fits. When this was not possible, gels were run in parallel as specified in the figure legend. Gels replicas on two independent experiments were performed for all experiments to qualitatively confirm reproducibility. Microscopy images were acquired in two different fields of view containing at least 20 droplets from two individual experiments, and representative images shown.
Data exclusions	No data were excluded.
Replication	All the results reported in the manuscript were successfully reproduced. Each experiment was performed at least twice. For qualitative microscopy, images were recorded in two different field of views from two individual experiments.
Randomization	n/a because we are not dealing with biological samples/organisms/participants
Blinding	n/a because we are not dealing with biological samples/organisms/participants

Reporting for specific materials, systems and methods

We require information from authors about some types of materials, experimental systems and methods used in many studies. Here, indicate whether each material, system or method listed is relevant to your study. If you are not sure if a list item applies to your research, read the appropriate section before selecting a response.

Materials & experimental systems

n/a	Involved in the study	
☒	☐	Antibodies
☒	☐	Eukaryotic cell lines
☒	☐	Palaeontology and archaeology
☒	☐	Animals and other organisms
☒	☐	Clinical data
☒	☐	Dual use research of concern

Methods

n/a	Involved in the study	
☒	☐	ChIP-seq
☒	☐	Flow cytometry
☒	☐	MRI-based neuroimaging
